# Use of the NIH consensus criteria in cellular and soluble biomarker research in chronic graft-versus-host disease: A systematic review

**DOI:** 10.3389/fimmu.2022.1033263

**Published:** 2022-10-25

**Authors:** Emina Milosevic, Antonija Babic, Lorenzo Iovino, Milos Markovic, Magdalena Grce, Hildegard Greinix

**Affiliations:** ^1^ University of Belgrade, Faculty of Medicine, Institute of Microbiology and Immunology, Belgrade, Serbia; ^2^ Department of Laboratory Immunology, Clinical Department of Laboratory Diagnostics, University Hospital Center Zagreb, Zagreb, Croatia; ^3^ Fred Hutchinson Cancer Center, Clinical Research Division, Seattle, WA, United States; ^4^ Division of Molecular Medicine, Ruđer Bošković Institute, Zagreb, Croatia; ^5^ Division of Haematology, Medical University of Graz, Graz, Austria

**Keywords:** chronic graft-versus-host disease, cellular and soluble biomarker, allogeneic hematopoietic cell transplantation, NIH consensus development project on criteria for clinical trials in cGvHD, CXCR3^+^CD56^bright^ NK cells, CD19^+^CD21^low^ B cells, BAFF/CD19^+^ B cell

## Abstract

**Objectives:**

Chronic graft-versus-host disease (cGvHD) is the most frequent cause of late non-relapse mortality after allogeneic haematopoietic stem cell transplantation (alloHCT). Nevertheless, established biomarkers of cGvHD are still missing. The National Institutes of Health (NIH) Consensus Development Project on Criteria for Clinical Trials in cGvHD provided recommendations for biomarker research. We evaluated to which extent studies on cellular and soluble biomarkers in cGvHD published in the last 10 years complied with these recommendations. Also, we highlight the most promising biomarker candidates, verified in independent cohorts and/or repeatedly identified by separate studies.

**Methods:**

We searched Medline and EMBASE for “cGvHD”, “biomarkers”, “soluble” and “cells” as MeSH terms or emtree subject headings, and their variations on July 28th, 2021, limited to human subjects, English language and last ten years. Reviews, case reports, conference abstracts and single nucleotide polymorphism studies were excluded. Criteria based on the set of recommendations from the NIH group for biomarker research in cGvHD were used for scoring and ranking the references.

**Results:**

A total of 91 references encompassing 15,089 participants were included, 54 prospective, 17 retrospective, 18 cross-sectional, and 2 studies included both prospective and retrospective cohorts. Thirty-five papers included time-matched controls without cGvHD and 20 studies did not have any control subjects. Only 9 studies were randomized, and 8 were multicentric. Test and verification cohorts were included in 11 studies. Predominantly, diagnostic biomarkers were explored (n=54). Assigned scores ranged from 5-34. None of the studies fulfilled all 24 criteria (48 points). Nevertheless, the scores improved during the last years. Three cell subsets (CXCR3^+^CD56^bright^ NK cells, CD19^+^CD21^low^ and BAFF/CD19^+^ B cells) and several soluble factors (BAFF, IL-15, CD163, DKK3, CXCL10 and the panel of ST2, CXCL9, MMP3 and OPN) had the highest potential as diagnostic and/or prognostic biomarkers in cGvHD.

**Conclusion:**

Despite several limitations of this review (limited applicability for paediatric population, definition of verification, missing data on comorbidities), we identified promising candidate biomarkers for further evaluation in multicentre collaborative studies. This review confirms the importance of the NIH consensus group criteria for improving the quality and reproducibility of cGvHD biomarker research.

## 1 Introduction

Chronic graft-versus-host disease (cGvHD) is a major complication and the main cause of non-relapse mortality (NRM) in patients after allogeneic haematopoietic cell transplantation (alloHCT) (1). This multi-system disease occurs in 30-70% of patients after alloHCT (2), negatively influencing their long-term outcomes, as well as their everyday quality of life (3). Known risk factors for cGvHD development are donor related ones including HLA mismatched or unrelated donors, older donor age, female donor for male recipient, mobilized peripheral blood stem cells (PBSC) as cell source and characteristics related to the patient such as older age, and previous acute GvHD (aGvHD) (4, 5).

Although the pathogenesis of cGvHD has not been completely elucidated yet, it is widely accepted that unique pathophysiologic mechanisms, different from those seen in aGvHD, may be operative in cGvHD. Based on available data, a three-step model has been proposed that includes the following phases: 1) early inflammation and tissue injury, 2) chronic inflammation and dysregulated immunity, and 3) aberrant tissue repair with fibrosis (6). The initial phase is characterized by the release of several inflammatory mediators and activation of the innate immune system due to cytotoxic drugs, infection and tissue damage caused by previous aGvHD. The second phase comprises activation of the adaptive immune system and disruption in central and peripheral tolerance pathways, resulting in immune dysregulation and chronic inflammation. This leads to the last phase, which includes aberrant tissue repair with the development of pathological fibrosis, manifested as excessive extracellular matrix deposition, and ensuing loss of normal tissue architecture and organ dysfunction (6). Such complex pathogenesis results in a whole spectrum of clinical manifestations with inflammatory and fibrotic features in single or multiple organs including cutaneous, oral, ocular, gastrointestinal (GI), musculoskeletal, genital manifestations and the involvement of the lymphohaematopoietic system (7). Due to its wide spectrum of clinical manifestations affecting many different organ systems, diagnosis of cGvHD has been a complex task that usually requires a multidisciplinary team of experts in different medical fields. The National Institutes of Health (NIH) consensus criteria include recommendations for diagnosis and severity scoring of cGvHD and have been in use in both clinical studies as well as routine patient assessments (8). This resulted in improved evaluation and documentation of cGvHD organ manifestations. Nonetheless, significant challenges in the treatment of cGvHD remain. Corticosteroids continue to be used for initial therapy. Short-term and long-term clinical response rates with corticosteroids are unsatisfactory (9), and prolonged administration causes significant side effects that can be as challenging as cGvHD itself. Thus, treatments of cGvHD are often ineffective, with frequent incomplete responses and cGvHD recurrences.

There is an urgent need for procedures and tools that would enable timely diagnosis of cGvHD, identify patients with high risk of disease progression, select treatment options based on pathophysiologic features in a given patient and predict therapeutic response. To this end, identification of biomarkers, defined as any characteristic that is objectively, accurately and reproducibly measured as an indicator of a certain pathogenic process and/or pharmacologic response to a therapeutic intervention (10), would be of utmost importance. In cGvHD, biomarkers could aid in the diagnosis of cGvHD (diagnostic biomarkers), cGvHD course (prognostic biomarkers), prediction of response to treatment before its initiation (predictive biomarkers) or to serve as surrogate endpoint during therapy (therapy response biomarkers). Various biological parameters have been proposed as potential biomarkers in cGvHD, such as genetic and epigenetic markers, metabolism products, alloantibodies, microbiota composition, extracellular vesicles, cells of certain phenotype, and soluble molecules (eg. cytokines) (11). Indeed, a number of different candidate biomarkers, which correlate with various cGvHD manifestations have been identified. However, biomarkers that could guide therapeutic decisions in cGvHD are still largely lacking. In order to transcend the gap between research and clinical applicability of biomarkers, the NIH Consensus Development Project on Criteria for Clinical Trials in Chronic Graft-versus-Host Disease proposed criteria and gave a framework for biomarker investigations that include identification, verification, qualification, and application of potential candidate biomarkers (12), but, at present, it is unclear how many studies aiming to identify biomarkers in cGvHD fulfil these rigorous criteria.

There are several excellent recent reviews on cGvHD biomarkers covering single nucleotide polymorphisms (SNPs) (13), epigenetic markers, extracellular vesicles, microbiota (11) and omics technologies regarding alloHCT outcomes (14) but none of them evaluated biomarker publications in light of study design, quality of reported patients’ data and used measurement methods. Therefore, one of the aims of the COST Action Integrated European Network on Chronic Graft Versus Host Disease (cGvHD) EUROGRAFT (CA17138) was to critically evaluate to which extent studies on cellular and soluble biomarkers in cGvHD published in the last 10 years complied with the NIH recommendations for biomarker research and to discuss which biomarkers are worthy of further investigation and verification/qualification based on NIH recommendations (12), available at: https://www.gvhd.eu/working-groups/working-group-2/). In this review, we focused on cellular and soluble diagnostic and prognostic biomarkers with the majority of them being immunological, established evaluation criteria based on the aforementioned NIH criteria and assessed peer-reviewed publications accordingly. Although biomarkers of response were beyond the scope of this review, we included these studies when the baseline biomarker measurements (i.e. before treatment) were available and related to cGvHD.

## 2 Materials and methods

### 2.1 Literature search

This is a systematic review conducted according to the guidelines provided by “The Preferred Reporting Items for Systematic reviews and Meta-Analyses (PRISMA) statement 2020”. A comprehensive literature search was undertaken using Medline (via PubMed) and EMBASE (via Ovid) for terms “cGvHD”, “biomarkers”, “soluble” and “cells”. We used MeSH terms and emtree subject headings, for PubMed and EMBASE, respectively, and variation of terms in spoken language (**Table 1**). The search was conducted on July 28^th^, 2021. Relevant references within each reviewed manuscript were scanned to identify other potentially relevant and eligible studies.

**Table 1:** Search strategy for Medline and EMBASE.

Database	Search query
Medline	((((((biological factor[MeSH Terms]) OR ((biomark*) OR biomarkers [MeSH Terms])))))) AND (((((((((chronic*) AND ((((((graft vs. host disease) OR graft vs host disease) OR graft-vs-host-disease) OR graft-versus-host-disease) OR graft versus host disease [MeSH Terms]) OR graft versus host disease*))) AND “english”[Language])) NOT (((single nucleotide polymorphism*) OR SNP*) OR single nucleotide polymorphism [MeSH Terms]))) AND ((((cell[MeSH Terms]) OR cell*) OR cells OR soluble))) AND “last 10 years”[PDat] AND Humans [Mesh]) NOT ((Case Reports [ptyp] OR Review [ptyp]))
EMBASE	1. biomarker.mp. or exp biological marker/ 2. exp chronic graft versus host disease/ 3. 1 and 2 4. limit 3 to (human and english language and last 10 years) 5. limit 4 to (article or article in press or conference paper or editorial or erratum or letter or note or short survey or tombstone)

### 2.2 Inclusion criteria

Prospective and retrospective studies with human subjects published in English language in the last ten years on cGvHD and soluble as well as cellular biomarkers were included. Reviews, single case reports and conference abstracts were excluded from this analysis. In addition, we excluded SNP studies since these were recently reviewed by Partanen et al. within the EUROGRAFT COST Action (13).

### 2.3 Study selection, quality assessment, and data extraction

**Figure 1:**
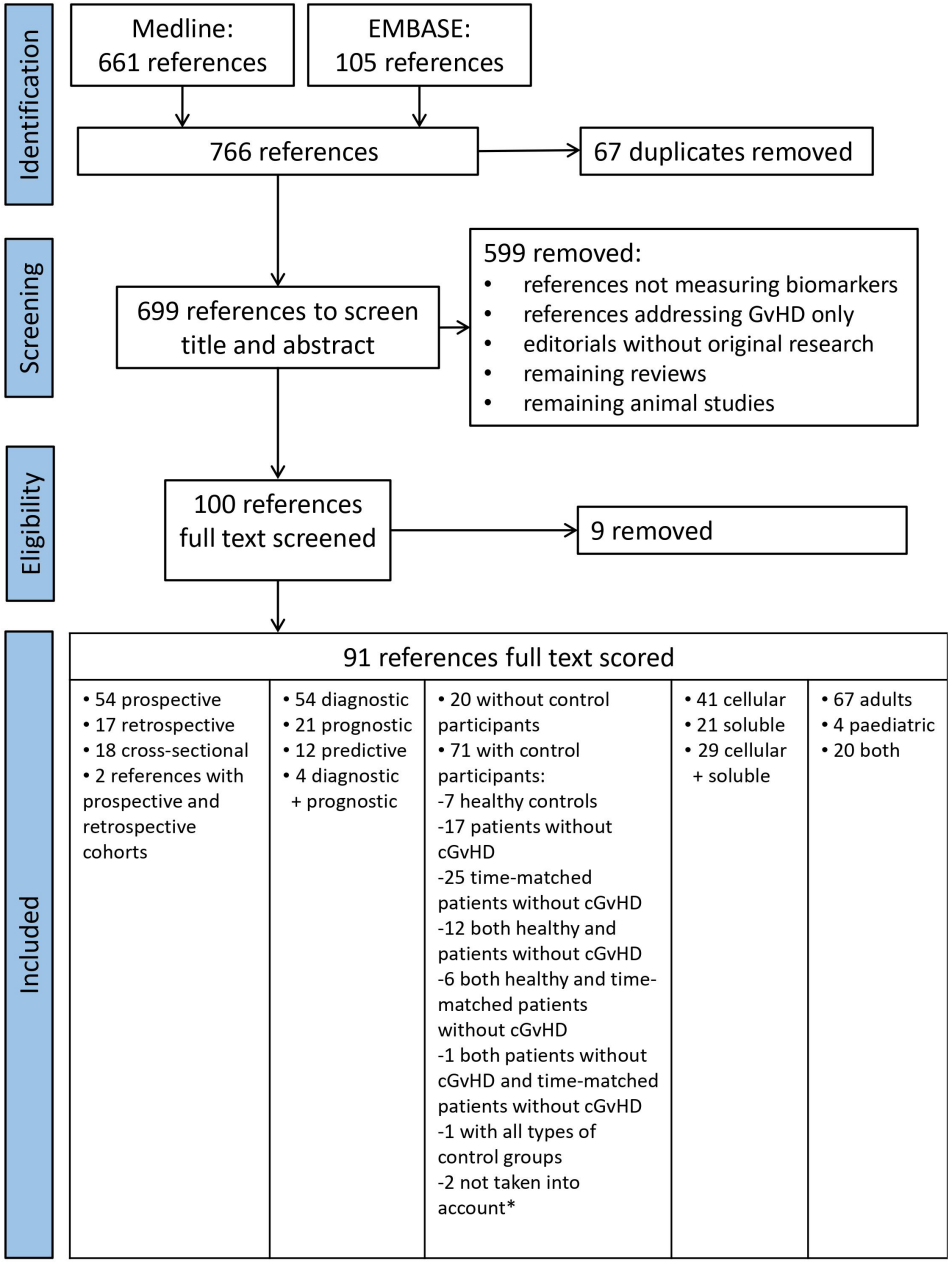
PRISMA Flow chart of literature search for cellular and soluble biomarkers of cGvHD. *Saad et al. (15) did not specify how many control subjects they had and Hirayama et al. (16) did an analysis of a subgroup of patients from their previous study.

Medline search retrieved 661 references and EMBASE search 105 ones (**Figure 1**). After duplicate removal, 699 non-overlapping references were initially screened randomly for relevance by title and abstract by four independent researchers (EM, AB, LI, and MM) whereby each paper was screened by at least two researchers. Papers without abstract (n=22) were also included and assessed in full text length in the first round. Disagreements among researchers were resolved by consensus after discussion with an expert (HG). In total, 599 references were excluded with the majority of them not reporting data on biomarker measurement. Reviews, editorials without original research data, interviews, animal studies without human subjects, and SNP studies that had remained after automatic search were also excluded. The resulting 100 references were further assessed as text in full length by at least 2 out of 5 independent researchers (EM, AB, LI, MM and HG) using a standardized electronic data extraction form. During that process additional 9 references were excluded since analyzed markers were not related to cGvHD leaving a total of 91 relevant references for data extraction.

Data were collected on patient characteristics, clinical outcomes, investigated cellular and soluble biomarkers and methodological quality of all included studies. The parameters extracted using a standardized form are shown in **Supplementary Table 1**. Based on the recommendations of the NIH Consensus Development Project on biomarker research in cGvHD (12) we included confounding factors such as presence of relapse of original disease or active infection, usage of steroid or other immunosuppressive drugs at biosampling, history of aGvHD, and details of cGvHD regarding its severity and duration. Furthermore, information on the presence of a time-matched alloHCT control without cGvHD, specification of time-points for biosampling and performance of serial analyses was included. In addition to these criteria, we also focused on technical details of investigated methods for biomarker analysis in included studies to allow comparisons and judgement on reproducibility of assays used.

### 2.4 Data analysis and statistical methods

Each of the 91 selected references was screened in full-length text by two researchers and scored applying the above-mentioned criteria and then ranked. To this end, a 0-2 range scale was used. Score of 2 (black circle in **Supplementary Table 2**) was assigned if the authors fully addressed the criterion; score of 1 (open circle) was assigned if the answer was partial; score of 0 (dash) if the criterion was not even addressed at all.

Score values were assessed for normality of distribution by Shapiro-Wilk’s test. Statistical significance of score differences across different types of studies was assessed by one-way ANOVA. Scores for subgroups of studies were compared by Student’s T test. Pearson’s correlation coefficient was used to assess correlations of scores with publication year. P values less than 0.05 were considered statistically significant. Statistical analysis was performed in IBM SPPS Statistics software ver. 22 (IBM Inc., Armonk, NY, USA).

## 3 Results

### 3.1 Identification of eligible studies

A full-text review of the selected 91 manuscripts revealed that the majority of studies was prospective (n=54, 54.6%), 17 were retrospective (17.2%), 18 cross-sectional (18.8%), while 2 studies had both prospective and retrospective cohorts (2.2%) included (**Figure 1**). They included a total of 15,089 subjects, among whom 5,828 patients had cGvHD and 4,435 were controls (416 healthy individuals, 4,019 were post-transplant patients without cGvHD, out of which 3,023 were time-matched for the time since alloHCT). The study from Saad et al. that included 2,736 participants, did not specify the absolute number of patients with and without cGvHD (15). The remaining 2,090 subjects had diseases other than cGvHD (e.g., aGvHD). There were 20 (22.0%) studies without any control subjects. Out of 71 papers with control subjects, one paper did not specify how many control subjects they had (15), and one paper was analysis of a subgroup of patients (16) from their previous study (17). These two papers (15, 16) were not taken into the analysis of a total number of papers with control subjects. The remaining 69 papers with control subjects included: 7 papers with healthy controls, 17 papers with a control group of patients without cGvHD, 25 papers with a control group of time-matched patients without cGvHD, 12 papers with a control group of healthy persons and patients without cGvHD, 6 papers with both healthy controls and time-matched patients without cGvHD, 1 paper with a control group of patients without cGvHD and a group of time-matched patients without cGvHD, and 1 study with all three groups of controls (patients without cGvHD, time-matched patients without cGvHD, and healthy controls). Among the selected 91 studies, 9 were randomized (9.9%) and 8 (8.8%) were multicentre studies. Test and verification cohort were included in 11 studies. Single time-point measurements were performed in 53 papers (58.2%), while 38 papers (41.8%) had serial measurements of biomarkers, respectively.

As for the type of biomarkers, diagnostic biomarkers were explored in 54 studies (59.3%), both diagnostic and prognostic in 4 studies (4.4%) and in 18 out of these 58 studies samples were obtained at first onset of cGvHD. Prognostic biomarkers were the focus of 21 papers (23.1%) including 8 with sample collection on day +100 after alloHCT, while 12 papers (13.2%) studied predictive biomarkers.Cellular markers were analyzed in 41 (45.0%) papers (5,856 patients), 21 papers (23.1%) assessed soluble markers (4,643 patients), while 29 papers (31.9%) included both soluble and cellular markers (4,174 patients) in the analysis.

Concerning the age of study participants, an adult population was assessed in 67 (73.6%) papers including 10,040 individuals, while 4 (4.4%) studies were performed in only paediatric populations and included 499 children. Mixed adult and paediatric cohorts were evaluated in 20 papers (22.0%) with a total number of 4,134 patients.

### 3.2 Adherence to the NIH criteria for biomarker identification/replication in cGvHD

**Figure 2:**
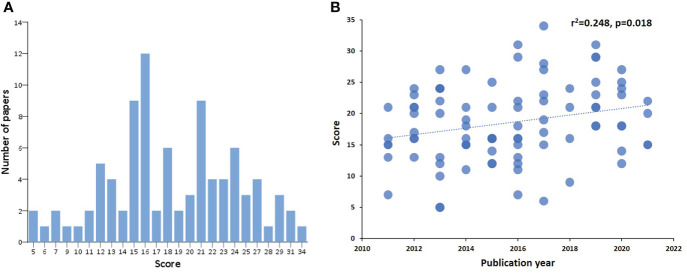
Score distribution of identified references and correlation with the year of publication. Score distribution of identified references **(A)**. The x-axis shows the score according to defined NIH criteria on biomarker research, and the y-axis represents the number of publications per score value. Correlation of score to year of publication **(B)**. On the x axis the year of publication can be seen, on the y axis the assigned score of each manuscript published in the respective year.

We scored 91 manuscripts according to a predefined list of criteria based on recommendations of the NIH consensus group on biomarker research in cGvHD regarding their adherence to these criteria (**Supplementary Tables 1, 2**). The scores followed normal distribution and ranged from 5-34 (the maximal possible score being 48, i.e. 2 points for each of 24 criteria) (**Figure 2A**). Mean score ± SD of all publications was 18.4 ± 6.1. Multicentre studies had a significantly higher mean score compared to single centre studies (24.7 ± 5.2 *vs.* 17.3 ± 5.6, p=0.000014), respectively. On the other hand, there were no significant differences in scores between prospective and retrospective studies. The scores significantly correlated with the year of publication and a trend of higher scores was observed in more recent publications (**Figure 2B**).

Information on time from alloHCT to sampling was provided by 79 of 91 (86.8%) papers as shown in **Supplementary Table 2**. Items most frequently reported include current immunosuppressive therapy (80.2%), current aGvHD (74.7%), and proper description of analysed biosamples (65.9%). Of note, NIH global severity score of cGvHD was only reported in 53 (58.2%) and time-point of sampling in 49 papers (53.8%), respectively. Least frequently reported were absence of cGvHD recurrence (12.1%), duration of cGvHD until sampling (14.3%) and time of onset of cGvHD (29.7%).

We critically assessed provided technical information only on flow cytometry, enzyme immunosorbent assay (ELISA) and quantitative polymerase chain reaction (qPCR). Authors provided sufficient data either directly or citing previous work (score 2) in 24 papers (26.4%), partial information about applied technique(s) (score 1) was given in 58 papers (63.7%), while nine studies (9.9%) did not give details on measurement techniques at all (score 0).

In this plethora of available studies, we tried to summarize data and propose the most promising biomarker candidates identified either in studies with test and verification cohort, or described by three or more independent studies as shown in **Table 2**. For the purpose of clarity, they are grouped as cellular and soluble biomarkers with the emphasis on their potential applicability as diagnostic and/or prognostic biomarker.

**Table 2:** Summary of most promising biomarkers of cGvHD.

Cells	Soluble factors	Type of biomarker	Reference
*Biomarkers confirmed in studies with a test and verification cohort*			
CXCR3^+^CD56^bright^ NK cells		diagnostic	(18)
	CXCL10	diagnostic	(18, 19)
	BAFF	diagnostic	(18)
	IL-15	diagnostic	(20)
	CD163	prognostic	(21)
	DKK3	diagnostic	(22)
	ST2, CXCL9, MMP3, OPN (as a panel)	diagnostic	(23)
*Biomarkers identified by at least 3 studies*			
CD19^+^CD21^low^ B cells		diagnostic	(24–30)
	CXCL10	diagnostic	(18, 19, 31)
	BAFF	diagnostic	(19, 28, 29, 32–37)
		prognostic	(36)
BAFF/CD19^+^ B cell ratio		diagnostic	(24, 28, 29, 32, 38)
		prognostic	(39)

BAFF, B cell activating factor; DKK, Dickkopf-related protein 3; ST2, suppression of tumorigenicity 2; MMP3, matrix metalloproteinase 3; OPN, osteopontin.

### 3.3 Cellular biomarkers of cGvHD

Chronic GvHD involves multiple, distinct interactions among alloreactive and dysregulated T and B cells and innate immune populations, including macrophages, dendritic cells (DCs), and neutrophils, that culminate in the initiation and propagation of profibrotic pathways. Both alloreactive effector cells as well as regulatory cell subsets that include, but are not limited to, regulatory T and B cells (Tregs and Bregs, respectively), and natural killer (NK) cells reportedly have been associated with different aspects of cGvHD, such as incidence, phenotypes, severity and outcomes, suggesting their possible role as biomarkers.

#### 3.3.1 B cells

Dysregulation in the B cell compartment is one of the most frequent findings associated with cGvHD. Significantly higher relative numbers of CD19^+^CD21^low^ B cells in patients with long-lasting or first diagnosis of cGvHD were reported by three independent research groups, including the highly scored publication by Lawitschka et al. with 146 paediatric patients with either cGvHD (n=38) or time-matched controls after alloHCT and a median follow-up of 8.6 years (24–30, 40, 41). Furthermore, CD19^+^CD21^low^ B cells significantly correlated with activity and severity of cGvHD (25, 26, 30, 42) and were elevated on day +100 after alloHCT in patients subsequently developing cGvHD (29). These studies had proper controls consisting of patients without cGvHD (**Table 2**). In a highly scored publication of Khoder et al. further characterized the CD19^+^CD21^low^ B cell subset demonstrating features of exhaustion, such as increased expression of multiple inhibitory receptors, altered expression of chemokine and adhesion molecules and poor proliferative response to a variety of stimuli (30). Regarding paediatric patients with cGvHD, Lawitschka et al. reported significantly higher CD19^+^CD21^low^ B cells (25) as had been observed in adult patients, whereas Schultz et al. surprisingly found lower CD19^+^CD21^low^ B cells in children with cGvHD (27). Both publications (25, 27) had the highest scores in our evaluation among papers assessing B cell biomarkers, and thoroughly documented their study design with description of patients and controls (children without cGvHD in both studies) in great detail. Lawitschka et al. (25) took biospecimens from paediatric patients a minimum of 100 days after alloHCT or suffering from ongoing cGvHD, previously resolved cGvHD and during early (until day +365 after alloHCT) and late (more than 365 days after alloHCT) follow-up whereas Schultz et al. (27) reported results on day +100 after alloHCT in children later developing cGvHD or not. Thus, analytical time points of these studies cannot be compared and a possible influence of patients’ age, and disease (malignant, non-malignant) as well as time after alloHCT and degree of immune reconstitution until biosampling cannot be assessed for certain and could have an impact on discrepancy of reported results. Furthermore, Schultz et al. report on biomarkers that predict risk of developing cGvHD whereas Lawitschka et al. analysed diagnostic biomarkers of cGvHD, respectively.

Another hallmark of cGvHD is the lower frequency of CD19^+^CD27^+^ memory B cells (25, 41) and their reduced number is associated with the incidence of infectious complications after alloHCT (26, 28, 41). Of note, resolution of cGvHD correlated with expansion of CD19^+^CD27^+^ memory B cells (25). Using B cell rearrangement excision circle measurements, Glauzy et al. observed an increased B cell replication but decreased overall B cell neogenesis in patients with aGvHD and cGvHD (32). Furthermore, they found a higher B cell division rate that correlated with an elevated BAFF/CD19^+^ B cell ratio in patients affected with cGvHD, supporting a B cell hyperactivation state *in vivo*. Activated CD27^+^ B cells from cGvHD patients reportedly were in an increased metabolic state with bigger size and protein content and were primed for survival *via* B cell activating factor (BAFF) pathways (43). Furthermore, B cells in cGvHD patients had significantly increased proliferative responses to B cell receptor (BCR) stimulation and after initiation of BCR signaling, B cells in cGvHD patients exhibited an increased B cell linker protein (BLNK) and Syk phosphorylation compared with B cells from patients without cGvHD (44). This effect could be mediated *via* increased BAFF levels. Thus, B cells have a lowered BCR signaling threshold in cGvHD associated with increased B cell proliferation and activation in response to antigen.

Compared to healthy individuals, cGvHD patients have decreased Breg cells defined as interleukin (IL)-10 secreting cells with reportedly different phenotypes. Khoder et al. defined B cells with immunoregulatory properties within both the CD19^+^IgM^+^CD27^+^ memory and CD19^+^CD24^high^CD38^high^ transitional B cell compartments and observed a lower frequency of Breg cells in patients with cGvHD (45). Furthermore, Breg cells in these patients were less likely to produce IL-10 compared to Bregs from healthy donors. De Masson et al. observed enriched IL-10 production in both the CD24^hi^CD27^+^ and CD27^hi^CD38^hi^ plasmablast B cell compartments (46). Patients with cGvHD had less CD24^hi^CD27^+^ B cells and IL-10–producing CD24^hi^CD27^+^ B cells and increased CD27^hi^CD38^hi^ plasmablast frequencies but decreased IL-10–producing plasmablasts (46).

In summary, among these various B cell phenotypes, elevated CD19^+^CD21^low^ B cells were the most consistent diagnostic biomarker candidate associated with cGvHD occurrence, activity and severity.

#### 3.3.2 Natural killer cells

In a highly scored manuscript in this review with adherence to important NIH criteria on biomarker research and the most vigorous design of a multicentre study with test, and verification cohorts, Kariminia et al. compared candidate biomarkers in adult patients with cGvHD with time-matched controls, and found that CXCR3^+^CD56^bright^ NK cells were significantly lower in cGvHD and had the closest inverse correlation to CXCL10 (18). These CD56^bright^ NK cells are known to exhibit an immunoregulatory role and are a classic NKreg population.

Cytolytic CD56^dim^ NK cells reportedly were increased on day +100 after alloHCT in paediatric patients subsequently developing cGvHD, a finding no longer observed in individuals of older age (27). Furthermore, noncytolytic CD56^bright^ regulatory NK cells were decreased in the cGvHD cohort (27). This NK cell subpopulation was also significantly decreased in peripheral blood (PB) at first diagnosis of cGvHD overall and even more pronounced in moderate/severe cGvHD, respectively (29).

The alloreactivity of NK cells is determined by various receptors including the activating CD94/NKG2C and the inhibitory CD94/NKG2A receptors, which both recognize the non-classical human leukocyte antigen E (HLA-E). NK cells expressing the activating CD94/NKG2C receptor were reduced in severe aGVHD and cGvHD (47), suggesting that a change in balance of activating and inhibitory receptors on NK cells could be a feature of cGvHD.

Briefly, CXCR3^+^CD56^bright^ regulatory NK cells could be a promising candidate for diagnostic marker of cGvHD.

#### 3.3.3 T cells

A number of studies evaluated usefulness of T cell phenotyping in diagnosis and prognosis of cGvHD. In a highly scored publication in this review on 241 paediatric patients analyzed on day 100 Schultz et al. reported increased percentages of activated CD3^+^CD69^+^ T cells, naïve CD4^+^CD45RA^+^CD31^-^helper T (Th) and cytotoxic CD8^+^CD45RA^+^PD1^+^ T cells in cGvHD compared to a control group without cGvHD (27). Faster reconstitution of CD4^+^ T cells and naïve CD4^+^ T cells at 1 month and CD8^+^ T cells at 3 months predicted more cGvHD, but better survival in recipients of G-CSF–mobilized PBSC grafts which was not the case in bone marrow (BM) recipients (48). When analyzed in the absence of active GvHD 3 months after alloHCT, patients subsequently developing *de novo* cGvHD had significantly higher relative and absolute counts of CD4^+^ T cells compared to patients with subsequent quiescent onset cGvHD and patients without cGvHD, respectively (49). In a prospective study with 163 adult cGvHD patients in comparison to time-matched alloHCT recipients who never experienced cGvHD, higher frequencies of CD4^+^CD45RA^+^CD31^+^ T cells assessed on day 100 after alloHCT were significantly associated with subsequent development of cGvHD and were also associated with diagnosis of cGvHD (29). Interestingly, Naeije et al. reported a more than 10-fold decrease in naïve CD4^+^CD45RA^+^ T cells and CD4^+^CD45RA^+^CD31^+^ T cells on day 100 in patients given anti-thymocyte globulin prophylaxis and an increase of these two T cell subsets in patients later developing cGvHD (50).

As for effector CD4^+^ T cell populations, Th1 and Th17 cells are regarded as pathogenic in cGvHD. CXCR3, which is considered a surrogate marker of Th1 cells, was shown to be highly expressed on infiltrating CD4^+^ T cells in tissue biopsies whereas an 80% decrease in CD4^+^ cells expressing CXCR3 was seen in the PB of cGvHD patients (31). In line with the presumed pathogenic role of Th17 cells, their frequency was significantly increased at cGvHD onset and drastically decreased after therapeutic response (51).

T follicular helper (Tfh) cells are a subset of T cells that provide essential signals to support germinal centers, memory B cells or high-affinity antibody producing plasma cell development (52). The frequency of functionally active ICOS^hi^PD-1^hi^ Tfh cells reportedly was increased in patients with active cGvHD compared with patients without cGvHD but was similar in patients with resolved cGvHD and no cGvHD and did not correlate with the clinical grade of cGvHD (53). Interestingly, this T cell subset decreased after rituximab treatment (42).

Among numerous Treg cell populations, the most of data are available on CD4^+^CD25^+^FoxP3^+^ T cells. In a top scored manuscript in this review Bohmann et al. found significantly higher relative and absolute Treg cell numbers in patients with *de novo* onset of cGvHD compared to those without or with quiescent onset of cGvHD (49). Studying the whole genome profile of CD4^+^CD25^hi^CD127^lo/-^ Treg cells at different time points identified defects on donor Treg cells associated with migration and suppressive function likely relevant for GvHD development (54). Expression profiles of interferon-induced proteins IFI44, IFIT3, IFI44L and IFIT1 increased after alloHCT with higher levels at onset of cGvHD. Activation and migration of Treg cells into tissue is critical in control of inflammation. FoxP3^+^ T cells increased in proportion to effector T cells in tissue infiltrates in oral and cutaneous lichenoid cGvHD, with a capacity to upregulate functional markers such as CD27, ICOS, and CD39 (55). Furthermore, functional markers and CXCR3 were both present in a higher proportion of FoxP3^+^ T cells in tissues than in PB, consistent with recruitment and activation of Treg cells in cGvHD target tissues. Furthermore, the proportion of resting CD45RA^+^FoxP3^+^ Treg cells in PB was significantly reduced in patients with lichenoid and sclerotic cGvHD as well as in the overall cGvHD cohort, compared with normal controls. This was also observed by Mahadeo et al. reporting decreased frequency and absolute numbers of resting CD4^+^CD127^-^CD25^+^CD45RA^+^FoxP3^+^ Treg cells in PB of paediatric patients with active cGvHD compared with healthy donors, alloHCT recipients without a history of cGvHD and recipients with resolved cGvHD (56).

Taken together, these studies imply the need for further assessment of defined T cell subpopulations in well-defined patient groups taking occurence of previous aGvHD, course of cGvHD and sampling time into consideration.

#### 3.3.4 Other cells

In a highly scored manuscript Lawitschka et al. described in a large paediatric patient cohort with underlying malignant diseases a significant association of increased monocyte numbers with active cGvHD compared to patients with no cGvHD (25). Furthermore, patients with NIH-defined severe cGvHD had a significantly higher monocyte number compared to mild and moderate cGvHD, respectively.

Patients with cGvHD tended to have significantly higher white blood counts, absolute neutrophils and platelet counts (57). As for the platelets, their higher counts were associated with active disease defined as clinician’s intention to intensify or alter systemic therapy due to the lack of response to current treatment. Likewise, higher platelet count was associated with more severe disease defined by NIH global score (57). Consistent with these findings, sclerotic form of cGvHD was associated with higher platelet counts (58). Thus, these results indicate the involvement of platelets in cGvHD biology, especially in genesis of sclerotic lesions, but their role as a biomarker of cGvHD is still not clear and warrants further research.

Mucosal associated invariant T (MAIT) cells stand at the interface of innate and adaptive immunity and are implicated in a broad range of infectious and non-infectious diseases. They are characterized by recognition of microbial riboflavin-derivative antigens and activation *via* T cell receptor-dependant and independent fashion (59). MAIT cells, identified as a subset of CD161^+^ T cells were found to be decreased in cGvHD (60). Another study showed reduced frequency of MAIT cells from CD4^-^, CD4^-^CD8^+^ and CD4^-^CD8^-^ T cell populations in mild cGvHD patients compared to patients without cGvHD and in patients with severe cGvHD compared to moderate cGvHD (38). These findings suggest an important role of MAIT cells, but more studies on these recently reported cells are needed regarding their biomarker potential.

### 3.4 Soluble factors as biomarkers of cGvHD

#### 3.4.1 BAFF

In a highly scored publication Kariminia et al. evaluated both previously known markers and performed discovery-based analysis for cGvHD biomarkers in two independent test sets selecting 11 markers that were further tested in two independent verification cohorts (18). They observed a high level of heterogeneity in cGvHD plasma biomarkers in a large cGvHD cohort with only soluble plasma B cell–activating factor (BAFF) and, more consistently, CXCL10 being the most reproducible markers. Therefore, the authors concluded that future analyses for plasma cGvHD biomarkers would need to be assessed for performance on very large patient groups with consideration of multiple covariates. Excessive levels of BAFF were found in patients with active cGvHD in multiple studies (19, 28, 29, 32–35). Significantly higher BAFF levels and BAFF/CD19^+^ B cell ratios were also reported in patients with newly diagnosed bronchiolitis obliterans syndrome (BOS) (24) and other patients with cGvHD compared with patients without cGvHD (28, 29, 32, 38). Furthermore, Glauzy et al. reported in patients with cGvHD prolonged lymphopenia and compensatory mechanisms leading to sustained B cell divisions up to 24 months after alloHCT that correlated with an elevated BAFF/CD19^+^ B-cell ratio, supporting a B cell hyperactivation state *in vivo* (32).

High BAFF levels in cGvHD patients were also associated with an inverse expression of BAFF-receptor (BAFF-R) on the surface of mature B cells (28, 33, 34) with elevated numbers of CD19^+^ CD27^−^CD10^−^CD21^low^ B cells and classical switched memory B cells, and reduced numbers of transitional and naïve B cells (28). Moreover, there is evidence that BAFF can also be used as a predictor of outcome, since plasma BAFF levels at diagnosis of cGvHD were significantly associated with NRM in a large study on 341 consecutive adult patients (36).

Patients on corticosteroid doses above 30 mg/day reportedly had significantly lower BAFF levels compared with those on lower corticosteroid doses or no steroid at all, a finding confirmed by others (29, 37). BAFF has been shown to be essential for B cell recovery after myeloablation as demonstrated in a prospective study monitoring 412 patients in the first year after alloHCT (39). In patients who did not develop cGvHD BAFF levels decreased as B cell numbers increased after myeloablative conditioning, which was not the case after reduced-intensity conditioning (RIC) with high BAFF levels throughout the first year after alloHCT. Furthermore, significantly higher BAFF/B cell ratios were observed at 3 months after alloHCT only after myeloablative conditioning in patients who subsequently developed cGvHD. In addition to alloHCT conditioning type, the use of sirolimus was significantly associated with higher BAFF levels after alloHCT (39). Therefore, the interpretation of BAFF levels after alloHCT should take these variables into account.

#### 3.4.2 Chemokines

Chemokines (e.g., CXCL9, CXCL10, CXCL11) are a large group of proteins that play an important role in the recruitment and retention of haematopoietic cells into specific tissues. This is particularly important in T cell migration, where the pattern of local chemokine expression and the profile of chemokine receptors on the cell surface determine the migration and retention of T cells within tissues. In the highest-ranking publication in this review, Abu Zaid et al. observed a significant association between high chemokine (C-X-C motif) ligand 9 (CXCL9) levels on day 100 after alloHCT and subsequent development of cGvHD in a prospective study on more than 200 patients (61). This finding has recently been confirmed by Orsatti et al. (62) in a cohort of 425 patients, including 190 without cGvHD as a control group.

CXCL9 and CXCL10 were analyzed in a large prospective study including independent test and verification cohorts for evaluation of cGvHD biomarkers performed by Kariminia et al. and highly scored in this review (18). Together with BAFF, CXCL10 was the only marker consistently associated with early diagnosis of cGvHD in both verification cohorts. In multivariate analysis, CXCL10 had an increased significance in combination with anti-LG3 and CXCL9, or inversely with CXCR3^+^CD56^bright^ NK cells (18). Croudace et al. observed significantly elevated serum concentrations of the CXCR3-binding chemokines, CXCL9, CXCL10, and CXCL11, in patients with active cGvHD of the skin compared to alloHCT patients without cGvHD (31). However, cGvHD patients without cutaneous involvement had no significant increase in chemokine levels. Others reported a significant increase of BAFF, CXCL10 and CXCL11 in serum of overall cGvHD patients (19). Yu et al. developed a panel of four lead plasma biomarkers consisting of suppression of tumorigenicity 2 (ST2), CXCL9, matrix metalloproteinase-3 (MMP-3), and osteopontin that significantly correlated with cGvHD diagnosis and was confirmed in a second verification cohort including samples from 8 different sites (23). Unfortunately, the association of biomarkers with cGvHD severity and NRM in the first verification cohort was not seen in the second one.

#### 3.4.3 Other pro-inflammatory and anti-inflammatory cytokines

As part of the BAFF system, another tumor necrosis factor (TNF) ligand superfamily member, a proliferation-inducing ligand (APRIL), was tested as a potential biomarker. APRIL shares two receptors with BAFF, transmembrane activator and calcium-modulator and cyclophilin ligand interactor (TACI) and B cell maturation antigen (BCMA), and is important in antibody class switching and plasma cell survival (63). In a prospective study on 73 patients, active cGvHD patients showed significantly higher APRIL levels compared with inactive ones. Moreover, APRIL correlated with plasmablast frequencies in the cGvHD subgroup, and high APRIL levels were associated with antinuclear antibody production and severe cGvHD (34). Furthermore, Chasset et al. observed a significantly decreased expression of BAFF-R and an increased expression of BCMA and TACI in patients with active cGvHD (34).

For a long time, an imbalance in cytokines that modulate inflammation has been supposed to contribute to the pathogenesis of cGvHD. A large amount of conflicting evidence in terms of cytokine and GvHD markers has been published. In a discovery cohort of 153 alloHCT recipients, 12 parameters were assessed revealing that patients with low levels of IL-15 on day 7 after alloHCT had a 2.7-fold higher likelihood of developing cGvHD in need of systemic immunosuppressive therapy than patients with higher IL-15 levels (20). This was confirmed in a verification cohort of 105 similarly treated patients demonstrating that patients with low IL-15 levels had a 3.7-fold higher likelihood of developing significant cGvHD. In a paediatric cohort of 170 alloHCT recipients a panel of six serum cytokines was monitored at established time points prior to and up to day 60 after alloHCT revealing that only higher IL-8 serum concentration on day +28 was significantly associated with a lower probability of cGvHD (64).

Yeh et al. serially analysed plasma levels after alloHCT and observed that IL-10 levels correlated with the clinical activity of GvHD and therapeutic responsiveness at all sites including gut, oral mucosa and liver (65). However, plasma IL-10 was not significantly different between aGvHD and cGvHD. IL-21 plasma levels reportedly were significantly increased in patients at onset of cGvHD, and day +30 plasma IL-21 levels were associated with severe cGvHD (51). Turcotte et al. evaluated donor serum and plasma concentrations of cytokines and adipokines from test (n=210) and verification (n=250) cohorts of matched, unrelated donor PBSC recipients with haematological malignancies identified through the Center for International Blood and Marrow Transplantation Research between 2000 and 2011 (66). In the test cohort a significant inverse association was identified between donor TNF concentrations and cGvHD, however these findings were not reproducible in the verification cohort.

In a highly scored publication, Inamoto et al. analysed plasma proteins in a discovery and verification proteomic study on a large patient number at 80+/- 14 days after alloHCT and observed that CD163, a macrophage scavenger receptor elevated in oxidative conditions was significantly higher in patients subsequently developing *de novo*-onset cGvHD (21).

Levels of pentraxin-3 (PTX3), an acute phase protein rapidly induced and secreted by diverse cell types in response to inflammation, were significantly lower in patients with cGvHD compared to alloHCT recipients without cGvHD and PTX3 levels correlated with severity of cGvHD (67).

#### 3.4.4 DKK3 and other soluble molecules

When comparing proteomic profiles between patients with newly diagnosed sclerotic cGvHD, those with newly diagnosed nonsclerotic cGvHD, and those without cGvHD using mass spectrometry analysis, Inamoto et al. identified Dickkopf-related protein 3 (DKK3), a modulator of the Wnt signaling pathway, as a biomarker for both sclerotic and nonsclerotic cGvHD (22). Verification analysis of 186 patients confirmed that elevated plasma DKK3 concentrations were associated with cGvHD, regardless of the presence or absence of sclerosis. Patients with high DKK3 plasma concentrations had a higher NRM than those with low concentrations. Akahoshi et al. observed that Mac-2 binding protein (M2BP) was significantly related to liver cGvHD but not to another organ involvement (68).

Grkovic et al. analyzed clinical markers of inflammation in the sera of patients with established cGvHD and correlated those with disease activity observing that lower albumin, higher C-reactive protein, and higher platelets were significantly associated with active disease (57). Patients with severe NIH global score had higher values of CRP, C3 and platelets compared to patients with moderate disease. Furthermore, a statistically significant association was found between higher levels of CRP, C3 and platelets, and more severe joint/fascia involvement (NIH score 3) and more severe skin involvement (NIH score 3). In a cross-sectional study evaluating 206 patients with cGvHD higher platelet count and C3 were also associated with sclerotic cGvHD (58).

Recent research has also been made into quantification of metabolites as potential novel biomarkers of cGvHD, suggesting cGvHD may be associated with expanded cellular energy and potentially mitochondrial dysfunction (69). These and other novel biomarkers were reviewed recently by EUROGRAFT COST action work group (11).

#### 3.4.5 Elafin and other organ-specific biomarker

Cutaneous involvement has been frequently seen in cGvHD and can be observed as lichenoid or sclerotic features. In a large-scale quantitative proteomic discovery study for identification of biomarker candidates of skin GvHD followed by the validation of the lead candidate, elafin, with enzyme-linked immunosorbent assay in samples from 492 patients, Paczesny et al. observed an overexpression of elafin in GvHD skin biopsies (70). Plasma concentrations of elafin were significantly higher at onset of skin GvHD, correlated with the eventual maximum grade of GvHD, and were associated with a greater risk of death relative to other known risk factors. Since only patients with skin rashes were analyzed they most likely had either aGvHD or overlap syndrome without documentation of cGvHD features.

Brüggen et al. analyzed skin biopsies from aGvHD, lichenoid and sclerotic cGvHD patients and detected no elafin in sclerotic cGvHD, whereas this molecule was increased in lichenoid cGvHD as compared with aGvHD (71). Elafin-high lichenoid cGvHD lesions presented with epidermal thickening and were associated with poor prognosis seen as corticosteroid resistance.

Upregulated Th1/Th17 cytokine/chemokine transcripts and elevated numbers of interferon (IFN)-γ– and IL-17–producing CD8^+^ T cells were found in skin of patients with lichenoid cGvHD compared to the ones with aGvHD (72). Furthermore, IL-17^+^ cells were identifed in 26/27 skin and in all gut and oral mucosa biopsies of patients with cGvHD, being more frequent in mucosa lesions than in the skin (73).

Salivary IL-10 and IL-6 levels correlated with severity of oral cGvHD (74). Devic et al. reported that among 249 salivary proteins identified by tandem mass spectrometry, 82 exhibited altered expression in the oral cGvHD patient group compared with patients without oral cGvHD (75). Two of these proteins, IL-1 receptor antagonist (IL-1Ra) and cystatin B, showed decreased expression in patients with active oral cGvHD and these two biomarkers were able to distinguish oral cGvHD with a sensitivity of 85% and specificity of 60%, respectively, and showed slightly better discrimination in newly diagnosed patients evaluated within 12 months of alloHCT.

Cocho et al. analysed tear levels of a panel of inflammatory molecules in ocular cGvHD patients and compared results to those in healthy subjects (76). Tear cytokine assessment revealed that levels of CXCL10 were significantly lower in tears of ocular cGvHD patients, positively correlating with tear production and negatively correlating with symptoms, hyperemia and vital staining. Among 19 cytokines assessed in tear fluid prior to alloHCT only the levels of the inflammatory molecules fractalkine, IL-1Ra, and IL-6 had good prognostic ability for the development of ocular cGvHD after alloHCT (77). Analysis of cytokines in tear fluid at onset of cGvHD revealed that tear IL-2, IL-10, IL-17A, IFN-γ, IL-6, and TNF were significantly elevated in patients with systemic cGvHD compared with patients without cGvHD (78). Tear cytokines IL-10, IL-6, and TNF showed a strong correlation with ocular surface parameters, such as staining scores, conjunctival injection and Schirmer’s test scores and these cytokines also correlated significantly with severity of ocular cGvHD.

#### 3.4.6 Relevant publications since performance of literature search and analysis

Recently, Inamoto et al. (79) observed significantly higher plasma concentrations of matrix metalloproteinase-9 (MMP-9) in patients with bronchiolitis obliterans syndrome (BOS) compared with those with non-BOS cGvHD or no cGvHD. MMP-3 concentrations were higher in patients with BOS or non-BOS cGvHD compared with those with no cGvHD, respectively. Furthermore, MMP-9 concentrations before treatment start were higher in patients who experienced treatment failure within 6 months compared with those with treatment success. High MMP-9 concentrations were also associated with worse overall survival, respectively. These findings of a discovery study, however, have to be confirmed by further research.

In two independent cohorts of a total of 289 cGvHD patients the proteomic plasma marker regenerating islet-derived protein 3-α (REG3α) was significantly increased in patients with GI cGvHD compared with those without (80). Patients with high REG3α had higher NRM and were 1.9 times more likely to die without relapse. These data warrant prospective biomarker verification studies.

## 4 Discussion

In 2015, the NIH consensus development project on cGvHD published a framework for biomarker investigations, consisting of identification, verification, qualification, and application with terminology based on Food and Drug Administration and European Medicines Agency guidelines (12). The main focus thereby was on diagnosis and assessment of cGvHD disease activity, prognostic risk to develop cGvHD and prediction of response to therapy. Furthermore, it was recommended that sample collection for cGvHD biomarker studies should be well documented following established quality control guidelines for sample acquisition, processing, preservation, and testing, at intervals that are both calendar and event driven. Moreover, standardized documentation of patients’ treatment should accompany biospecimen analyses. To date, no cGvHD biomarkers have been qualified for use in clinical applications yet, although an increasing number of cGvHD candidate biomarkers have been reported and thus, are available for further investigation.

In this extensive review we evaluated publications on cGvHD biomarker research of the last ten years regarding their compliance with the NIH recommendations. This is, to our knowledge, the first systematic evaluation of this kind. We identified 91 manuscripts investigating various cellular and soluble biomarkers of cGvHD and scored them according to a predefined checklist of NIH recommended items. In addition, publications were assessed regarding reproducibility of reported biomarker results in included independent verification patient cohorts. Remarkably, none of the publications included in this review met all of the 24 criteria used and the highest score was 34 out of a maximum of 48. Notably, the mean score of all publications was 18.4. The NIH global severity score of cGvHD was only reported in about half of the publications and the least frequently described items were duration of cGvHD until sampling, time of onset of cGvHD and whether patients experienced cGvHD recurrence. Thus, important information for proper interpretation of biomarker results was not available and this lack of clinical data could have an impact on reproducibility of biomarker results by other investigators. Multicentre studies had a significantly higher mean score compared to single centre studies indicating thorough discussions and considerations of study design and trial conduct in a larger group of researchers, more experience in performing trials and better infrastructure for study conduct. Most promisingly, we show that the scores significantly correlated with the year of publication and higher scores were observed in more recent manuscripts although a correlation coefficient of 0.248 has considered to be weak. Despite this fact our analysis demonstrates very well the acceptance of the NIH consensus document on biomarker research in the cGvHD community despite the high stringency of these criteria.

Paczesny et al. describe as initial step for the identification of candidate biomarkers analyses in a small patient cohort with well-matched cases and appropriate controls and a clear definition of the clinical context of biomarker use to allow proper collection of supporting clinical data to assess a clinical endpoint (12). In this review, a multitude of biomarker discovery analyses is included and only about half of all had controls at all and 35% had time-matched controls. This shows quite well that there is a need to improve detailed description of study designs for cGvHD biomarker identification to obtain meaningful results with a clear benefit for clinicians in decision making.

To confirm the analytical validity of a test the NIH consensus requests as second step after identification of a potential biomarker analyses to demonstrate the test’s reproducibility and accuracy as well as its practicality and cost-effectiveness. In the current review, only 11 of 91 identified studies (12.1%) had test and verification cohorts. The highly scored multicentre study of Kariminia et al. in adult patients with cGvHD with time-matched controls included test and validation cohorts and subsequently performed biomarker verification in two independent patient groups (18). Thereby, they observed that CXCR3^+^CD56^bright^ NK cells were significantly lower in cGvHD patients and had the closest inverse correlation to CXCL10. To our knowledge, this cell subpopulation known to exhibit an immunoregulatory role (81) is the only cellular biomarker in cGvHD that has been verified, so far.

In addition to scoring, we also propose a summary of the most promising biomarkers in cGvHD defined as reproducible ones as shown in verification studies and/or those biomarkers repeatedly identified by at least three different research groups. Beside the CXCR3^+^CD56^bright^ NK cells, these include the CD19^+^CD21^low^ B cell subpopulation, since significantly higher CD19^+^CD21^low^ B cells have been reported by many investigators in patients with first diagnosis as well as long-lasting cGvHD, respectively (24–30, 40, 41). Not too surprisingly more soluble biomarkers, namely BAFF, CXCL10, IL-15, CD163, DKK3; and the panel of ST2, CXCL9, MMP3 and OPN, were reported as soluble biomarker with evidence of reproducibility as seen in verification analyses (18–23). Future studies will have to confirm the usefulness of these soluble biomarkers in clinical practice.

We are aware of potentially important limitations of this review. First, biomarker studies were not reviewed separately for different age groups. This was due to the fact that only 4 (4.4%) studies reported results in paediatric populations including a total of 499 children (25, 27, 56, 82). Furthermore, studies in adult cGvHD patients assessed a broad age range of patients and most of the time did not report results separately according to age cohorts. Given the fact that time to immune reconstitution differs in younger and older individuals (83) and immunological markers can be impacted not only by cGvHD but also by age-associated changes of the immune system (84), future studies should draw patients’ age into consideration. Second, data on paediatric immune reconstitution regarding the various underlying malignant and non-malignant diseases are limited. Lawitschka et al. assessed cellular and humoral parameters of immune reconstitution in a large paediatric cohort with early and long-term follow-up allowing insights into the disturbance of immune reconstitution by the occurrence and persistence of cGvHD as well as differences between patients with and without underlying malignant diseases (25). Although it is well-known that prolonged immunodeficiency after alloHCT is associated with severe infectious complications (41, 85, 86), also in adult patient populations, most biomarker studies did not have indicators of immune reconstitution, such as naïve T cells and memory B cells included as confounding variables in their analyses. Third, we considered confirmation of biomarker findings with the same technology in an independent patient cohort as fulfilling a test’s reproducibility and accuracy and thus, called this to be a verified biomarker knowing that the NIH consensus definition included also a test’s practicality and cost-effectiveness in their definition of verification (12). It was, however, beyond the scope of this review to analyze the later in more detail. Fourth, very few studies referred to comorbidities of alloHCT recipients, such as obesity or diabetes mellitus that could affect the cellular composition and function of cells of the innate and adaptive immune system as well (87–89).

## 5 Conclusions

In the recent NIH consensus meeting on criteria for clinical trials in cGvHD, participants emphasized the importance of uniform protocols for sampling and processing of biospecimens and recommended the verification of previously identified biomarkers in well-defined clinical study protocols (90). Based on the results included in this manuscript it is fair to conclude that much work will be required to verify and qualify the candidate biomarkers identified so far. Close collaboration between multi-specialized clinical and laboratory-based groups will be needed to pursue studies that will subsequently lead to clinical application of much needed diagnostic and prognostic biomarkers in cGvHD. The present review underlines the importance of the NIH consensus group criteria in order to improve the quality and reproducibility of cGvHD biomarker research.

## Data availability statement

The original contributions presented in the study are included in the article/**Supplementary Material**. Further inquiries can be directed to the corresponding author.

## Author contributions

EM and HG conceptualized the manuscript. EM, AB, LI, and MM drafted the manuscript. HG critically revised the manuscript and MG contributed to the modification and revision of the manuscript. All authors contributed to the article and approved the submitted version.

## Funding

The work on this review was supported by COST Action Integrated European Network on Chronic Graft Versus Host Disease (cGvHD) EUROGRAFT, CA17138.

## Acknowledgments

The authors would like to acknowledge the members of the EUROGRAFT COST Action Working group on molecular and cellular biomarkers of cGvHD: Anne Dickinson, Marit Inngjerdingen, Daniel Wolff, Drazen Pulanic, Atillio Olivieri, Robert Zeiser, Rachel Crossland, Nuala Mooney, Ralf Dressel, Sara Galimberti, Francesca Perutelli, Antoine Toubert, Jukka Partanen, Kati Hyvärinen, Matteo Doglio, Ewa Karakulska-Prystupiuk, Günther Eißner, Katarzyna Bogunia-Kubik, Marie Lipoldova, Milena Ivanova, Serap Evran, Philippe Lewalle, Lars Klingen Gjærde, Georg Stary, Antonio Perez Martinez, Maja Pucic-Bakovic, and Nina Milutin Gasperov.

## Conflict of interest

The authors declare that the research was conducted in the absence of any commercial or financial relationships that could be construed as a potential conflict of interest.

## Publisher’s note

All claims expressed in this article are solely those of the authors and do not necessarily represent those of their affiliated organizations, or those of the publisher, the editors and the reviewers. Any product that may be evaluated in this article, or claim that may be made by its manufacturer, is not guaranteed or endorsed by the publisher.
