# Supplementary material for: Use of the NIH consensus criteria in cellular and soluble biomarker research in chronic graft-versus-host disease: A systematic review
Source: Front Immunol. 2022 Oct 25;13:1033263. doi: 10.3389/fimmu.2022.1033263 (PMC9641232; doi:10.3389/fimmu.2022.1033263)
Supplement: Supplementary file 1 [file Table_1.docx]

**Supplementary table 1.** Scoring criteria

|  | Criterion | Explanation |
| --- | --- | --- |
| 1) | Time from alloHCT to sampling | Described sampling time relative to alloHCT |
| 2) | Absence of cGvHD recurrence | Authors state whether they included or excluded patients with recurrence of cGvHD in the analysis |
| 3) | Absence of relapse of the underlying malignancy | Authors state whether they included or excluded patients with relapse of their underlying malignancy in the analysis |
| 4) | Absence or presence of recurrent or ongoing aGvHD | Authors state whether they included or excluded patients with recurrent aGvHD or aGvHD progressing into cGvHD |
| 5) | Absence or presence of active infection | Authors state whether they included or excluded patients with active infection at the time of sampling |
| 6) | CMV serostatus | Check whether CMV serostatus or active CMV infection was assessed or not |
| 7) | Absence of recent B cell depletion after alloHCT | Authors state whether they included or excluded patients recently undergoing B cell depletion prior to sampling |
| 8) | Manipulation or treatment of the donor product | A note given on whether the donor product was manipulated (i.e., TCD, G-CSF) or not |
| 9) | T or B cell depletion during conditioning | A note on whether the patient underwent T or B cell depletion during conditioning is given and how it was addressed in the analysis |
| 10) | Current immunosuppressive therapy | A note on whether the patient had immunosuppressive therapy at the time of sampling (e.g., corticosteroids, CNI) and how it was addressed in the analysis |
| 11) | NIH global severity score | Severity of cGvHD is scored according to the NIH global severity score |
| 12) | Randomized study | Note on whether the study was randomized |
| 13) | Control cohort | Note on whether the biomarker was measured in a group of healthy people or group without cGvHD |
| 14) | Time-matched controls without cGvHD | A patient cohort without cGvHD was time-matched to a cGvHD group or not |
| 15) | Severity of cGvHD | Assessment whether cGvHD severity was scored by a scale other than the NIH score |
| 16) | Duration of cGvHD | Duration of cGvHD at the time of sampling |
| 17) | Analyzed material is described properly | Stated if anticoagulant is used, whether the whole blood/isolated cells were used, fresh or frozen cells were analyzed, etc. |
| 18) | Specified time-point of sampling | Time of sampling (after alloHCT) with a timeframe of deviation of this point is specified by study design |
| 19) | Serial analyses performed | At least two measurement points in the study |
| 20) | Documented minimization of bias in patient selection | Patient selection is explained and made clear how the bias is addressed |
| 21) | Time of onset of cGvHD | Time of onset of cGvHD after alloHCT |
| 22) | Multicenter study | Study included several centers that performed biomarker measurements themselves (i.e., if samples from several centers were sent to a central laboratory for analysis, it was not scored as multicenter study) |
| 23) | Test and replication/validation cohort | Study design included biomarker assessment in another independent cohort of patients |
| 24) | Enzyme immunosorbent assay  Flow cytometry  qPCR | Detailed description of the ELISA (or other) kit – product name and/or catalogue number, manufacturer stated  Stated antibody clones, antibody concentrations, labelling fluorophores, instrument used for acquisition, gating strategy, acquisition and analysis software version  Primer and probe sequences, labelling, concentrations or assay catalogue name and number, manufacturer, PCR cycling conditions, instrument, software version |

Abbreviations: alloHCT, allogeneic haematopoietic stem cell transplantation; cGvHD chronic graft-versus-host disease; aGvHD, acute graft-versus-host disease; CMV, cytomegalovirus; TCD, T cell depletion; G-CSF, granulocyte colony-stimulating factor; CNI, calcineurin inhibitors; NIH, National Institutes of Health; ELISA, enzyme-linked immunosorbent assay; qPCR, quantitative polymerase chain reaction
