# Supplementary material for: Use of the NIH consensus criteria in cellular and soluble biomarker research in chronic graft-versus-host disease: A systematic review
Source: Front Immunol. 2022 Oct 25;13:1033263. doi: 10.3389/fimmu.2022.1033263 (PMC9641232; doi:10.3389/fimmu.2022.1033263)
Supplement: Supplementary file 2 [file Table_2.docx]

**Supplementary table 2**. Graphic representation of references’ scoring

| Reference* | | Time from alloHSCT to sampling | Absence of cGvHD relapse | Absence of relapse of an underlying malignancy | Absence or presence of current or recent aGvHD | Absence or presence of active infection | CMV serostatus | Absence of recent B cell depletion after alloHSCT | Manipulation or treatment of the donor product | T or B cell depletion during conditioning | Current immune suppressive therapy | NIH global severity score | Randomized study | Control cohort (healthy, no cGvHD) | Time-matched controls without cGvHD | Severity of cGvHD– other than NIH | Duration of cGvHD | Analyzed material is described properly | Specified time-point of sampling | Serial analyses performed | Documented minimization of bias in the patient selection | Time of onset of cGvHD | Multicenter study | Test and validation cohort | Measurement techniques | SCORE |
| --- | --- | --- | --- | --- | --- | --- | --- | --- | --- | --- | --- | --- | --- | --- | --- | --- | --- | --- | --- | --- | --- | --- | --- | --- | --- | --- |
| Abu Zaid *et al.* (1) | | ● | ● | ● | ● | ● | ● | - | - | ● | ● | - | ● | ● | - | - | - | ● | ● | ● | ● | ● | ● | - | ● | 34 |
| Kariminia *et al.* (2) | | ● | ● | - | ● | ● | ● | - | - | ● | ● | ● | - | - | ● | - | ● | ● | ● | - | - | ● | ● | ● | ○ | 31 |
| Lawitschka *et al.* (3) | | ● | ● | ● | ● | ○ | - | ● | ○ | ● | - | ● | - | ● | - | ● | ● | ○ | ● | ● | ○ | ● | - | - | ○ | 31 |
| Akahoshi *et al.* (4) | | ● | - | ● | ● | - | ● | - | - | ● | ● | ● | ● | ● | ● | - | - | ● | ● | ● | - | - | - | ● | ○ | 29 |
| Arai *et al.* (5) | | ● | - | ● | ● | ● | - | ● | - | - | ● | ● | ● | - | - | ● | ● | ● | - | - | ● | ● | ● | - | ○ | 29 |
| Waller *et al.* (6) | | ● | - | ● | ● | - | - | - | ● | ● | ● | - | ● | - | ● | - | - | ● | ● | ● | ● | ● | ● | - | ○ | 29 |
| Malard *et al.* (7) | | ● | ● | - | ● | ● | ○ | ● | - | ● | ● | ● | - | - | - | - | - | ● | ● | ● | - | ● | ● | - | ○ | 28 |
| Inamoto *et al.* (8) | | ● | ● | ● | ● | - | - | ● | - | ● | ● | ● | - | ● | ● | - | - | ● | ● | - | - | - | - | ● | ○ | 27 |
| Jacobson *et al.* (9) | | ● | - | ● | ● | - | - | ● | ● | - | ● | ● | - | - | ● | ● | - | ● | ○ | ● | ○ | ● | - | - | ○ | 27 |
| Pratt *et al.* (10) | | ● | - | ● | ● | ● | - | - | - | ● | ● | ● | - | - | ● | ● | - | ● | ● | ● | - | - | - | ● | ○ | 27 |
| Schultz *et al.* (11) | | ● | - | ● | ● | ● | - | - | - | ● | ● | ● | - | - | ● | - | - | ● | ● | ● | - | ● | ● | - | ○ | 27 |
| Ahmed *et al.* (12) | | ● | - | - | ● | - | - | - | - | - | ● | - | ● | ● | ● | - | - | ● | ● | ● | ● | - | ● | ● | ○ | 25 |
| Inamoto *et al.* (13) | | - | ● | - | ● | ● | - | ● | - | - | ● | ● | ● | - | ● | - | - | ● | - | ● | ● | - | - | ● | ○ | 25 |
| Jang *et al.* (14) | | ● | - | ● | ● | ● | ● | - | ● | ● | ● | - | - | - | ● | - | - | ● | ● | ● | - | - | - | - | ○ | 25 |
| Berger *et al.* (15) | | ● | - | ● | ● | - | ● | - | ● | - | ● | - | - | - | ● | ○ | - | ● | ● | ● | - | ● | - | - | ○ | 24 |
| Grossekatthofer *et al.* (16) | | ● | - | - | ● | ● | ● | - | ● | - | ● | - | - | - | ● | ● | - | ● | ● | ● | - | - | - | - | ● | 24 |
| Kuzmina *et al.* (17) | | ● | ○ | ● | - | ○ | - | ○ | - | - | ● | ● | - | - | ● | ● | - | ● | ● | ● | ○ | - | - | - | ● | 24 |
| Khoder *et al.* (18) | | ● | - | ● | ● | - | - | ● | - | ● | ● | ● | ● | ● | - | ● | - | ○ | ○ | - | - | - | - | - | ● | 24 |
| Yeh *et al.* (19) | | ● | ● | ● | ● | ● | - | - | - | - | ● | - | - | - | ● | ● | - | ○ | ● | ● | - | ● | - | - | ○ | 24 |
| Penack *et al.* (20) | | - | - | ● | ● | ● | ● | - | - | ● | ● | ● | - | - | - | - | - | ● | ● | - | ● | - | ● | - | ● | 24 |
| Cho *et al.* (21) | | ● | - | ● | ● | ● | ● | - | - | ● | ● | - | - | - | ● | - | - | ● | ● | ● | - | - | - | - | ○ | 23 |
| Klimczak *et al.* (22) | | ● | - | ● | ● | ○ | - | - | ● | ● | ● | ● | - | ● | - | - | - | ● | - | - | - | ● | - | - | ● | 23 |
| Naeije *et al.* (23) | | ● | - | - | ● | - | - | - | ● | ● | - | ● | ● | - | ● | - | - | ● | ● | - | ● | - | ● | - | ○ | 23 |
| Bohmann *et al.* (24) | | ● | - | ● | ● | ● | - | ● | - | - | ● | - | - | - | ● | ● | ○ | ● | - | - | - | ● | - | - | ○ | 22 |
| Doehn *et al.* (25) | | ● | - | - | ● | ○ | ● | - | - | - | ● | ● | - | ● | - | - | - | ● | ● | ● | - | ● | - | - | ○ | 22 |
| Zhao *et al.* (26) | | ● | ● | - | ● | - | - | - | - | ● | ● | ● | - | ● | ● | ● | ○ | - | ● | - | - | - | - | - | ○ | 22 |
| Yong *et al.* (27) | | ● | - | ● | ● | - | ● | - | - | ● | ● | ● | - | ● | - | - | - | - | - | ● | ● | - | - | - | ● | 22 |
| Saliba *et al.* (28) | | ● | - | ● | ● | - | - | - | - | ● | ● | ● | - | - | - | - | - | ● | ● | ● | - | ● | - | ● | ○ | 21 |
| Croudace *et al.* (29) | | ● | - | - | ● | - | - | ● | - | - | ● | - | - | - | ● | ● | ● | ● | ● | - | - | ● | - | - | ○ | 21 |
| Glauzy *et al.* (30) | | ● | - | - | ● | - | - | ● | - | ● | ● | - | - | - | ● | - | - | ● | ● | ● | - | ● | - | - | ○ | 21 |
| Sarantopoulos *et al.* (31) | | ● | - | ● | ● | - | - | ● | ● | - | ● | - | - | - | - | - | - | ● | ● | - | ● | ● | - | - | ○ | 21 |
| Ukena *et al.* (32) | | ● | - | - | ● | ● | - | - | - | ● | - | - | - | - | ● | ○ | - | ○ | ● | ● | ● | ● | - | - | ○ | 21 |
| Greinix *et al.* (33) | | ● | - | - | ● | ○ | - | - | - | - | ● | ● | - | - | ● | - | - | ● | ● | ● | ○ | - | ● | - | ○ | 21 |
| Du *et al.* (34) | | ● | - | ● | ● | - | - | - | - | - | ● | ● | - | - | ● | - | - | ● | ● | - | - | ● | - | ● | ○ | 21 |
| Turcotte *et al.* (35) | | - | - | ● | ● | - | - | ● | ● | ● | ● | - | - | - | - | - | - | ● | - | - | ● | - | ● | ● | ○ | 21 |
| Yu *et al.* (36) | | ● | - | ● | ● | - | - | - | - | - | ● | ● | - | - | ● | - | - | ● | ● | ● | - | - | - | ● | ○ | 21 |
| Bolanos-Meade *et al.* (37) | | ● | ● | - | ● | - | ● | - | - | - | ● | - | ● | - | - | - | - | - | ● | ● | ● | - | ● | - | - | 20 |
| Ezzat *et al.* (38) | | ● | - | ● | ● | - | - | - | - | ● | ● | ● | - | - | - | - | - | ● | ● | - | ● | - | - | - | ● | 20 |
| Perz *et al.* (39) | | ● | - | - | ● | - | ● | - | - | ● | ● | ● | - | ● | - | - | - | ● | ● | - | - | - | - | - | ● | 20 |
| Turcotte *et al.* (40) | | ● | - | ● | ● | - | ● | - | - | ● | - | - | - | ● | - | - | - | - | ● | ● | ● | - | - | ● | ○ | 19 |
| Bruggen *et al.* (41) | | ● | - | - | ● | - | - | - | - | - | ● | ● | - | - | - | ● | - | ● | ● | ● | - | ● | - | - | ○ | 19 |
| Metafuni *et al.* (42) | | ● | - | ● | ● | - | - | - | - | - | ● | - | - | ● | - | - | ● | ● | ● | ● | - | - | - | - | ○ | 19 |
| Rozmus *et al.* (43) | | ● | - | - | ● | - | ● | - | - | - | ● | - | - | ● | ● | - | ● | ○ | - | - | - | ● | - | - | ○ | 18 |
| Goklemez *et al.* (44) | | ● | ● | - | - | - | - | - | - | ● | ● | ● | - | ● | - | ● | ● | ● | - | - | - | - | - | - | - | 18 |
| Matos *et al.* (45) | | ● | - | ● | ● | - | - | - | ● | ● | - | - | - | ● | - | - | - | ● | ● | - | - | - | - | - | ● | 18 |
| Mrazek *et al.* (46) | | ● | - | - | ● | - | - | - | - | - | ● | ● | - | - | ● | - | - | ● | ● | ● | - | - | - | - | ● | 18 |
| Saad *et al.* (47) | | - | - | ● | ● | - | - | ● | ● | ● | ● | - | - | - | - | - | - | ● | - | - | ● | - | ● | - | - | 18 |
| Stikvoort *et al.* (48) | | ○ | - | - | ● | - | - | - | - | ● | ● | ● | - | ● | - | - | - | ○ | ○ | ● | - | - | - | - | ● | 17 |
| van der Waart *et al.* (49) | | ● | - | - | ● | - | - | - | ● | - | - | - | - | ● | - | ● | - | ○ | ● | ● | - | - | - | - | ● | 17 |
| Khoder *et al.* (50) | | ● | - | - | ● | - | - | ● | - | - | ● | ● | - | ● | - | - | - | ● | ○ | - | - | - | - | ● | ○ | 16 |
| Aki *et al.* (51) | | ● | - | ● | ● | - | - | - | - | - | ● | ● | - | ● | - | - | - | ● | ● | - | - | - | - | - | - | 16 |
| Allen *et al.* (52) | | ● | ○ | - | ● | - | - | ● | - | ● | ● | - | - | - | - | - | - | ● | - | - | - | - | ● | - | ○ | 16 |
| Bruggen *et al.* (53) | | ● | - | - | ● | - | - | - | - | - | ● | ● | - | ● | - | ● | - | ● | - | - | - | - | - | - | ● | 16 |
| Cocho *et al.* (54) | | - | - | ● | - | ● | - | - | - | - | ● | - | - | ● | - | ○ | ● | ● | - | - | - | ● | - | - | ○ | 16 |
| Devic *et al.* (55) | | ● | - | - | ● | - | - | - | - | - | ● | ● | - | ● | - | - | - | ● | ○ | - | - | - | - | ● | ○ | 16 |
| Fedoriw *et al.* (56) | | ● | - | - | ● | - | - | - | - | - | ● | - | - | ● | - | - | - | ● | ● | - | - | ● | - | - | ● | 16 |
| Gleimer *et al.* (57) | | ● | - | ● | ● | ● | ○ | - | - | - | ● | - | - | - | - | - | - | ● | ● | - | - | - | - | - | ○ | 16 |
| Knorr *et al.* (58) | | ● | - | - | ● | - | - | - | - | ○ | ○ | ● | - | - | ● | - | - | ○ | ● | ● | - | - | - | - | ○ | 16 |
| Kuzmina *et al.* (59) | | ● | - | - | ● | ● | - | - | - | - | ● | ● | - | - | - | ● | ● | ○ | - | - | - | - | - | - | ○ | 16 |
| Kuzmina *et al.* (60) | | ● | ○ | - | - | - | - | ● | - | - | ● | ● | - | - | - | - | ● | ○ | - | - | - | ● | - | - | ● | 16 |
| Motta *et al.* (61) | | ● | ○ | - | ● | - | - | - | - | - | ● | ● | - | ● | - | - | - | - | ○ | ● | - | - | - | - | ● | 16 |
| Tumino *et al.* (62) | | ● | - | - | ● | ● | ● | - | - | - | - | - | - | ● | - | - | - | ○ | ● | ● | - | - | - | - | ○ | 16 |
| Allen *et al.* (63) | | ● | - | - | ● | - | - | ● | - | ● | ● | - | - | ● | - | - | - | ● | - | - | - | - | - | - | ○ | 15 |
| Budde *et al.* (64) | | ● | - | - | ● | - | - | - | - | - | ● | ● | - | ● | - | - | - | ● | ● | - | - | - | - | - | ○ | 15 |
| Babić *et al.* (65) | | ● | - | - | ● | - | - | - | - | - | ● | ● | - | - | - | - | ● | ● | - | - | - | ● | - | - | ○ | 15 |
| Cocho *et al.* (66) | | - | - | ● | ● | - | - | - | - | - | ● | - | - | ● | - | ○ | - | ● | ● | - | - | ○ | - | - | ○ | 15 |
| Fingrut *et al.* (67) | | ● | - | ● | - | ● | - | - | - | - | ● | ● | - | ● | - | - | - | ● | - | - | - | - | - | - | ○ | 15 |
| Imanguli *et al.* (68) | | ● | - | - | - | - | - | - | - | - | ● | ● | - | ● | - | ● | ● | ○ | - | - | - | - | - | - | ● | 15 |
| Whittle *et al.* (69) | | - | - | ● | - | ● | - | - | - | - | ● | ● | - | - | - | - | - | ● | ● | ● | - | - | - | - | ○ | 15 |
| Mahadeo *et al.* (70) | | - | - | - | ● | - | - | - | - | ● | ○ | ● | - | ● | - | - | - | ● | - | - | ● | - | - | - | ● | 15 |
| Martires *et al.* (71) | | ● | ● | - | ● | - | - | - | - | - | ○ | ● | - | ● | - | - | ● | - | - | - | - | ● | - | - | - | 15 |
| Jung *et al.* (72) | | ● | - | - | - | ● | - | - | - | - | ● | ● | - | - | ● | ○ | - | ● | - | - | - | - | - | - | ○ | 14 |
| Lynch Kelly *et al.* (73) | | ● | - | ● | - | - | - | - | - | - | ● | ● | - | ● | - | - | - | ● | - | - | - | - | - | - | ● | 14 |
| Grkovic *et al.* (74) | | ○ | - | - | - | ○ | - | - | - | - | ● | ● | - | ● | - | ● | - | ○ | - | - | - | ● | - | - | - | 13 |
| Kordelas *et al.* (75) | | ● | - | ● | ● | - | - | - | - | - | - | - | - | - | ● | - | - | ○ | ● | ● | - | - | - | - | - | 13 |
| Marcondes *et al.* (76) | | ● | - | - | ● | - | ● | - | - | - | ● | - | - | ● | - | - | - | ● | - | - | - | - | - | - | ○ | 13 |
| Moins-Teisserenc *et al.* (77) | | ● | - | ● | - | - | - | - | - | - | - | - | - | ● | ● | - | - | ● | ● | - | - | - | - | - | ○ | 13 |
| de Masson *et al.* (78) | | ● | ○ | - | - | - | - | - | - | - | ● | ● | - | ● | - | - | - | ● | - | - | - | - | - | - | ○ | 12 |
| Hirayama *et al.* (79) | | ○ | - | - | - | ● | - | - | - | - | ● | ● | - | ● | - | - | - | - | - | ● | - | - | - | - | ○ | 12 |
| Konuma *et al.* (80) | | ● | - | ● | - | - | - | - | - | - | ● | ● | - | ● | - | - | - | ○ | - | - | - | - | - | - | ○ | 12 |
| Leonard *et al.* (81) | | - | - | ● | ● | ○ | ○ | - | - | - | ● | ● | - | - | - | - | - | - | - | - | - | - | - | - | ● | 12 |
| Sarvaria *et al.* (82) | | ● | - | - | - | - | - | - | - | - | - | - | - | ● | ● | - | - | ○ | ● | ● | - | - | - | - | ○ | 12 |
| Botari *et al.* (83) | | ● | - | - | - | - | - | - | - | - | ● | - | - | ● | ● | - | - | ○ | - | - | - | - | - | - | ● | 11 |
| Durakovic *et al.* (84) | | ● | - | - | - | - | - | - | - | - | ● | ● | - | ● | - | - | - | ● | - | - | - | - | - | - | ○ | 11 |
| Eberwein *et al.* (85) | | ● | - | - | - | - | - | - | - | - | ● | - | - | ● | - | - | - | ● | - | - | - | - | - | - | ● | 10 |
| Konuma *et al.* (86) | | ○ | - | - | - | - | - | - | - | - | ○ | ● | - | ● | - | - | - | ○ | - | - | - | - | - | - | ● | 9 |
| Chasset *et al.* (87) | | - | - | - | - | - | - | - | - | - | ● | ● | - | ● | - | - | - | - | - | - | - | - | - | - | ○ | 7 |
| Lai *et al.* (88) | | - | - | - | - | - | - | - | - | - | ○ | - | - | ● | - | - | - | ○ | - | - | - | - | - | ● | ○ | 7 |
| Hu *et al.* (89) | | ○ | - | - | ● | - | - | - | - | - | - | - | - | ● | - | - | - | - | - | - | - | - | - | - | ○ | 6 |
| Hirayama *et al.* (90) | | ○ | - | - | - | ● | - | - | - | - | - | - | - | ● | - | - | - | - | - | - | - | - | - | - | - | 5 |
| St John *et al.* (91) | | - | - | - | - | - | - | - | - | - | ○ | - | - | - | - | - | - | - | ● | ● | - | - | - | - | - | 5 |
| Papers fully addressing the criterion | **n** | **73** | **11** | **40** | **68** | **23** | **16** | **17** | **12** | **31** | **73** | **53** | **9** | **49** | **32** | **18** | **13** | **60** | **49** | **38** | **16** | **27** | **14** | **11** | **24** | / |
|  | **%** | **80.2** | **12.1** | **44.0** | **74.7** | **25.3** | **17.6** | **18.7** | **13.2** | **34.1** | **80.2** | **58.2** | **9.9** | **53.8** | **35.2** | **19.8** | **14.3** | **65.9** | **53.8** | **41.8** | **17.6** | **29.7** | **15.4** | **12.1** | **26.4** | / |

● Criterion fully addressed (2 points assigned)

○ Criterion incompletely covered, or not clear how it was addressed in the analysis (1 point assigned)

- Criterion not addressed (0 points assigned)

For example: score 1 was assigned if the presence of CMV infection and/or CMV serostatus was mentioned, but it was not clearly stated how it was taken into account in the analysis, or, patient sub-classification according to CMV serostatus/infection was not shown. Similarly, score 1 was assigned if ELISA was mentioned in the Methods section, but the exact ELISA kit used was not specified (no catalog number or full product name), or, when flow cytometry results were presented without precise gating strategy or monoclonal antibody clone(s) used. (In case the authors referred to methods explained in another paper, that paper was analyzed and the same criteria applied.) Of note, for criteria/questions that could be answered only with yes or no, score 2 or 0 were used, respectively.

*Reference numbering in this table differs from the main text, thereof the appropriate list of references is provided here.

Abbreviations: alloHCT, allogeneic haematopoietic stem cell transplantation; cGvHD chronic graft-versus-host disease; aGvHD, acute graft-versus-host disease; CMV, cytomegalovirus; NIH, National Institutes of Health

**References**

1. Abu Zaid M, Wu J, Wu C, Logan BR, Yu J, Cutler C, et al. Plasma Biomarkers of Risk for Death in a Multicenter Phase 3 Trial with Uniform Transplant Characteristics Post-Allogeneic Hct. *Blood* (2017) 129(2):162-70. Epub 2016/11/10. doi: 10.1182/blood-2016-08-735324.

2. Kariminia A, Holtan SG, Ivison S, Rozmus J, Hebert MJ, Martin PJ, et al. Heterogeneity of Chronic Graft-Versus-Host Disease Biomarkers: Association with Cxcl10 and Cxcr3+ Nk Cells. *Blood* (2016) 127(24):3082-91. Epub 2016/03/30. doi: 10.1182/blood-2015-09-668251.

3. Lawitschka A, Gueclue ED, Januszko A, Körmöczi U, Rottal A, Fritsch G, et al. National Institutes of Health-Defined Chronic Graft-Vs.-Host Disease in Pediatric Hematopoietic Stem Cell Transplantation Patients Correlates with Parameters of Long-Term Immune Reconstitution. *Frontiers in immunology* (2019) 10:1879. Epub 2019/09/12. doi: 10.3389/fimmu.2019.01879.

4. Akahoshi Y, Nakasone H, Kawamura K, Kusuda M, Kawamura S, Takeshita J, et al. Increased Mac-2 Binding Protein Glycan Isomer in Patients at Risk for Late Nonrelapse Mortality after Hsct. *Blood advances* (2019) 3(21):3287-96. Epub 2019/11/08. doi: 10.1182/bloodadvances.2019000629.

5. Arai S, Pidala J, Pusic I, Chai X, Jaglowski S, Khera N, et al. A Randomized Phase Ii Crossover Study of Imatinib or Rituximab for Cutaneous Sclerosis after Hematopoietic Cell Transplantation. *Clinical cancer research : an official journal of the American Association for Cancer Research* (2016) 22(2):319-27. Epub 2015/09/18. doi: 10.1158/1078-0432.ccr-15-1443.

6. Waller EK, Logan BR, Fei M, Lee SJ, Confer D, Howard A, et al. Kinetics of Immune Cell Reconstitution Predict Survival in Allogeneic Bone Marrow and G-Csf-Mobilized Stem Cell Transplantation. *Blood advances* (2019) 3(15):2250-63. Epub 2019/07/28. doi: 10.1182/bloodadvances.2018029892.

7. Malard F, Labopin M, Yakoub-Agha I, Chantepie S, Guillaume T, Blaise D, et al. Rituximab-Based First-Line Treatment of Cgvhd after Allogeneic Sct: Results of a Phase 2 Study. *Blood* (2017) 130(20):2186-95. Epub 2017/09/03. doi: 10.1182/blood-2017-05-786137.

8. Inamoto Y, Martin PJ, Paczesny S, Tabellini L, Momin AA, Mumaw CL, et al. Association of Plasma Cd163 Concentration with De Novo-Onset Chronic Graft-Versus-Host Disease. *Biology of blood and marrow transplantation : journal of the American Society for Blood and Marrow Transplantation* (2017) 23(8):1250-6. Epub 2017/04/30. doi: 10.1016/j.bbmt.2017.04.019.

9. Jacobson CA, Sun L, Kim HT, McDonough SM, Reynolds CG, Schowalter M, et al. Post-Transplantation B Cell Activating Factor and B Cell Recovery before Onset of Chronic Graft-Versus-Host Disease. *Biology of blood and marrow transplantation : journal of the American Society for Blood and Marrow Transplantation* (2014) 20(5):668-75. Epub 2014/01/28. doi: 10.1016/j.bbmt.2014.01.021.

10. Pratt LM, Liu Y, Ugarte-Torres A, Hoegh-Petersen M, Podgorny PJ, Lyon AW, et al. Il15 Levels on Day 7 after Hematopoietic Cell Transplantation Predict Chronic Gvhd. *Bone marrow transplantation* (2013) 48(5):722-8. Epub 2012/11/21. doi: 10.1038/bmt.2012.210.

11. Schultz KR, Kariminia A, Ng B, Abdossamadi S, Lauener M, Nemecek ER, et al. Immune Profile Differences between Chronic Gvhd and Late Acute Gvhd: Results of the Able/Pbmtc 1202 Studies. *Blood* (2020) 135(15):1287-98. Epub 2020/02/13. doi: 10.1182/blood.2019003186.

12. Ahmed SS, Wang XN, Norden J, Pearce K, El-Gezawy E, Atarod S, et al. Identification and Validation of Biomarkers Associated with Acute and Chronic Graft Versus Host Disease. *Bone marrow transplantation* (2015) 50(12):1563-71. Epub 2015/09/15. doi: 10.1038/bmt.2015.191.

13. Inamoto Y, Martin PJ, Lee SJ, Momin AA, Tabellini L, Onstad LE, et al. Dickkopf-Related Protein 3 Is a Novel Biomarker for Chronic Gvhd after Allogeneic Hematopoietic Cell Transplantation. *Blood advances* (2020) 4(11):2409-17. Epub 2020/06/04. doi: 10.1182/bloodadvances.2020001485.

14. Jang JE, Hwang DY, Chung H, Kim SJ, Eom JI, Jeung HK, et al. Early Cytomegalovirus Reactivation and Expansion of Cd56(Bright)Cd16(Dim/-)Dnam1(+) Natural Killer Cells Are Associated with Antileukemia Effect after Haploidentical Stem Cell Transplantation in Acute Leukemia. *Biology of blood and marrow transplantation : journal of the American Society for Blood and Marrow Transplantation* (2019) 25(10):2070-8. Epub 2019/06/19. doi: 10.1016/j.bbmt.2019.06.008.

15. Berger M, Signorino E, Muraro M, Quarello P, Biasin E, Nesi F, et al. Monitoring of Tnfr1, Il-2rα, Hgf, Ccl8, Il-8 and Il-12p70 Following Hsct and Their Role as Gvhd Biomarkers in Paediatric Patients. *Bone marrow transplantation* (2013) 48(9):1230-6. Epub 2013/04/16. doi: 10.1038/bmt.2013.41.

16. Grosekatthofer M, Guclu ED, Lawitschka A, Matthes-Martin S, Mann G, Minkov M, et al. Ferritin Concentrations Correlate to Outcome of Hematopoietic Stem Cell Transplantation but Do Not Serve as Biomarker of Graft-Versus-Host Disease. *Annals of Hematology* (2013) 92(8):1121-8. doi: <http://dx.doi.org/10.1007/s00277-013-1737-x>.

17. Kuzmina Z, Krenn K, Petkov V, Körmöczi U, Weigl R, Rottal A, et al. Cd19(+)Cd21(Low) B Cells and Patients at Risk for Nih-Defined Chronic Graft-Versus-Host Disease with Bronchiolitis Obliterans Syndrome. *Blood* (2013) 121(10):1886-95. Epub 2013/01/11. doi: 10.1182/blood-2012-06-435008.

18. Khoder A, Alsuliman A, Szydlo R, Apperley JF, Cooper N, Basar R, et al. Evidence for B Cell Exhaustion in Chronic Graft-Versus-Host Disease. *Frontiers in Immunology* (2018) 8(JAN):1937. doi: <http://dx.doi.org/10.3389/fimmu.2017.01937>.

19. Yeh SP, Liao YM, Lo WJ, Lin CL, Bai LY, Lin CY, et al. Kinetics of T Helper Subsets and Associated Cytokines Correlate Well with the Clinical Activity of Graft-Versus-Host Disease. *PLoS One* (2012) 7(9):e44416. Epub 2012/09/08. doi: 10.1371/journal.pone.0044416.

20. Penack O, Peczynski C, van der Werf S, Finke J, Ganser A, Schoemans H, et al. Association of Serum Ferritin Levels before Start of Conditioning with Mortality after Allosct - a Prospective, Non-Interventional Study of the Ebmt Transplant Complications Working Party. *Frontiers in immunology* (2020) 11:586. Epub 2020/05/01. doi: 10.3389/fimmu.2020.00586.

21. Cho BS, Lim JY, Yahng SA, Lee SE, Eom KS, Kim YJ, et al. Circulating Il-17 Levels During the Peri-Transplant Period as a Predictor for Early Leukemia Relapse after Myeloablative Allogeneic Stem Cell Transplantation. *Annals of hematology* (2012) 91(3):439-48. Epub 2011/09/07. doi: 10.1007/s00277-011-1318-9.

22. Klimczak A, Suchnicki K, Sedzimirska M, Lange A. Diverse Activity of Il-17(+) Cells in Chronic Skin and Mucosa Graft-Versus-Host Disease. *Archivum immunologiae et therapiae experimentalis* (2019) 67(5):311-23. Epub 2019/06/10. doi: 10.1007/s00005-019-00549-2.

23. Naeije L, Kariminia A, Abdossamadi S, Azadpour S, Subrt P, Kuzeljevic B, et al. Anti-Thymocyte Globulin Prophylaxis Induces a Decrease in Naive Th Cells to Inhibit the Onset of Chronic Graft-Versus-Host Disease: Results from the Canadian Bone Marrow Transplant Group (Cbmtg) 0801 Study. *Biology of blood and marrow transplantation : journal of the American Society for Blood and Marrow Transplantation* (2020) 26(3):438-44. Epub 2019/11/23. doi: 10.1016/j.bbmt.2019.11.015.

24. Bohmann EM, Fehn U, Holler B, Weber D, Holler E, Herr W, et al. Altered Immune Reconstitution of B and T Cells Precedes the Onset of Clinical Symptoms of Chronic Graft-Versus-Host Disease and Is Influenced by the Type of Onset. *Annals of hematology* (2017) 96(2):299-310. Epub 2016/12/13. doi: 10.1007/s00277-016-2881-x.

25. Doehn JM, Winkler A, Kuzmina Z, Hladik A, Greinix H, Knapp S, et al. Pentraxin-3 Levels in Graft-Versus-Host Disease During Allogeneic Hematopoietic stem Cell Transplantation. *Experimental hematology* (2016) 44(10):917-23. Epub 2016/07/13. doi: 10.1016/j.exphem.2016.07.002.

26. Zhao XY, Lv M, Xu LL, Qian X, Huang XJ. Donor Th17 Cells and Il-21 May Contribute to the Development of Chronic Graft-Versus-Host Disease after Allogeneic Transplantation. *European journal of immunology* (2013) 43(3):838-50. Epub 2013/01/03. doi: 10.1002/eji.201242816.

27. Yong X, Peng Y, Liu Z, Li Q, Lai Y, Wu T, et al. Analysis of Serum and Salivary Cytokines among Patients with Oral Cgvhd after Allo-Hsct. *Oral diseases* (2021) 27(5):1320-4. Epub 2020/10/03. doi: 10.1111/odi.13658.

28. Saliba RM, Sarantopoulos S, Kitko CL, Pawarode A, Goldstein SC, Magenau J, et al. B-Cell Activating Factor (Baff) Plasma Level at the Time of Chronic Gvhd Diagnosis Is a Potential Predictor of Non-Relapse Mortality. *Bone marrow transplantation* (2017) 52(7):1010-5. Epub 2017/05/10. doi: 10.1038/bmt.2017.73.

29. Croudace JE, Inman CF, Abbotts BE, Nagra S, Nunnick J, Mahendra P, et al. Chemokine-Mediated Tissue Recruitment of Cxcr3+ Cd4+ T Cells Plays a Major Role in the Pathogenesis of Chronic Gvhd. *Blood* (2012) 120(20):4246-55. Epub 2012/09/27. doi: 10.1182/blood-2012-02-413260.

30. Glauzy S, Soret J, Fournier I, Douay C, Moins-Teisserenc H, Peffault de Latour R, et al. Impact of Acute and Chronic Graft-Versus-Host Disease on Human B-Cell Generation and Replication. *Blood* (2014) 124(15):2459-62. Epub 2014/09/04. doi: 10.1182/blood-2014-05-573303.

31. Sarantopoulos S, Stevenson KE, Kim HT, Washel WS, Bhuiya NS, Cutler CS, et al. Recovery of B-Cell Homeostasis after Rituximab in Chronic Graft-Versus-Host Disease. *Blood* (2011) 117(7):2275-83. Epub 2010/11/26. doi: 10.1182/blood-2010-10-307819.

32. Ukena SN, Geffers R, Buchholz S, Stadler M, Franzke A. Biomarkers for Acute and Chronic Graft-Versus-Host Disease in Regulatory T Cells. *Transplant immunology* (2012) 27(4):179-83. Epub 2012/08/15. doi: 10.1016/j.trim.2012.07.003.

33. Greinix HT, Kuzmina Z, Weigl R, Körmoczi U, Rottal A, Wolff D, et al. Cd19+Cd21low B Cells and Cd4+Cd45ra+Cd31+ T Cells Correlate with First Diagnosis of Chronic Graft-Versus-Host Disease. *Biology of blood and marrow transplantation : journal of the American Society for Blood and Marrow Transplantation* (2015) 21(2):250-8. Epub 2014/12/03. doi: 10.1016/j.bbmt.2014.11.010.

34. Du J, Flynn R, Paz K, Ren HG, Ogata Y, Zhang Q, et al. Murine Chronic Graft-Versus-Host Disease Proteome Profiling Discovers Ccl15 as a Novel Biomarker in Patients. *Blood* (2018) 131(15):1743-54. Epub 2018/01/20. doi: 10.1182/blood-2017-08-800623.

35. Turcotte LM, Wang T, Hemmer MT, Spellman SR, Arora M, Yingst A, et al. Proinflammatory Cytokine and Adipokine Levels in Adult Unrelated Marrow Donors Are Not Associated with Hematopoietic Cell Transplantation Outcomes. *Biology of blood and marrow transplantation : journal of the American Society for Blood and Marrow Transplantation* (2019) 25(1):12-8. Epub 2018/08/26. doi: 10.1016/j.bbmt.2018.08.011.

36. Yu J, Storer BE, Kushekhar K, Abu Zaid M, Zhang Q, Gafken PR, et al. Biomarker Panel for Chronic Graft-Versus-Host Disease. *Journal of clinical oncology : official journal of the American Society of Clinical Oncology* (2016) 34(22):2583-90. Epub 2016/05/25. doi: 10.1200/jco.2015.65.9615.

37. Bolaños-Meade J, Wu J, Logan BR, Levine JE, Ho VT, Alousi AM, et al. Lymphocyte Phenotype During Therapy for Acute Graft-Versus-Host Disease: A Brief Report from Bmt-Ctn 0302. *Biology of blood and marrow transplantation : journal of the American Society for Blood and Marrow Transplantation* (2013) 19(3):481-5. Epub 2012/12/19. doi: 10.1016/j.bbmt.2012.12.003.

38. Ezzat S, Lam W, Law AD, Pasic I, Michelis FV, Kim DDH, et al. Prolactin, a Potential Biomarker for Chronic Gvhd Activity. *European Journal of Haematology* (2021) 106(2):158-64. doi: <http://dx.doi.org/10.1111/ejh.13531>.

39. Perz JB, Gürel S, Schonland SO, Hegenbart U, Ho AD, Dreger P. Cd4+Cd25highcd127low Regulatory T Cells in Peripheral Blood Are Not an Independent Factor for Chronic Graft-Versus-Host Disease after Allogeneic Stem Cell Transplantation. *TheScientificWorldJournal* (2012) 2012:606839. Epub 2012/06/06. doi: 10.1100/2012/606839.

40. Turcotte LM, Cao Q, Cooley SA, Curtsinger J, Holtan SG, Luo X, et al. Monocyte Subpopulation Recovery as Predictors of Hematopoietic Cell Transplantation Outcomes. *Biology of blood and marrow transplantation : journal of the American Society for Blood and Marrow Transplantation* (2019) 25(5):883-90. Epub 2019/01/10. doi: 10.1016/j.bbmt.2019.01.003.

41. Brüggen MC, Klein I, Greinix H, Bauer W, Kuzmina Z, Rabitsch W, et al. Diverse T-Cell Responses Characterize the Different Manifestations of Cutaneous Graft-Versus-Host Disease. *Blood* (2014) 123(2):290-9. Epub 2013/11/21. doi: 10.1182/blood-2013-07-514372.

42. Metafuni E, Giammarco S, De Ritis DG, Rossi M, Corrente F, Piccirillo N, et al. Changes in Protein Serum Levels During Stem Cell Transplantation. *European journal of clinical investigation* (2017) 47(10):711-8. Epub 2017/08/11. doi: 10.1111/eci.12796.

43. Rozmus J, Kariminia A, Abdossamadi S, Storer BE, Martin PJ, Lee SJ, et al. Comprehensive B Cell Phenotyping Profile for Chronic Graft-Versus-Host Disease Diagnosis. *Biology of blood and marrow transplantation : journal of the American Society for Blood and Marrow Transplantation* (2019) 25(3):451-8. Epub 2018/11/18. doi: 10.1016/j.bbmt.2018.11.007.

44. Goklemez S, Im AP, Cao L, Pirsl F, Steinberg SM, Curtis LM, et al. Clinical Characteristics and Cytokine Biomarkers in Patients with Chronic Graft-Vs-Host Disease Persisting Seven or More Years after Diagnosis. *American journal of hematology* (2020) 95(4):387-94. Epub 2020/01/07. doi: 10.1002/ajh.25717.

45. Matos TR, Hirakawa M, Alho AC, Neleman L, Graca L, Ritz J. Maturation and Phenotypic Heterogeneity of Human Cd4+ Regulatory T Cells from Birth to Adulthood and after Allogeneic Stem Cell Transplantation. *Frontiers in immunology* (2020) 11:570550. Epub 2021/02/05. doi: 10.3389/fimmu.2020.570550.

46. Mrazek F, Schneiderova P, Kriegova E, Raida L, Kuba A, Gajdos P, et al. Profile of Inflammation-Associated Proteins in Early Post-Transplant Samples of Patients after Allogeneic Hematopoietic Stem Cell Transplantation: A Preliminary Study. *Archivum immunologiae et therapiae experimentalis* (2016) 64(Suppl 1):55-61. Epub 2017/01/14. doi: 10.1007/s00005-016-0446-2.

47. Saad A, Lamb L, Wang T, Hemmer MT, Spellman S, Couriel D, et al. Impact of T Cell Dose on Outcome of T Cell-Replete Hla-Matched Allogeneic Peripheral Blood Stem Cell Transplantation. *Biology of blood and marrow transplantation : journal of the American Society for Blood and Marrow Transplantation* (2019) 25(9):1875-83. Epub 2019/05/16. doi: 10.1016/j.bbmt.2019.05.007.

48. Stikvoort A, Sundberg B, Chen Y, Lakshmikanth T, Mikes J, Radestad E, et al. Combining Flow and Mass Cytometry in the Search for Biomarkers in Chronic Graft-Versus-Host Disease. *Frontiers in Immunology* (2017) 8(JUN):717. doi: <http://dx.doi.org/10.3389/fimmu.2017.00717>.

49. van der Waart AB, van der Velden WJ, van Halteren AG, Leenders MJ, Feuth T, Blijlevens NM, et al. Decreased Levels of Circulating Il17-Producing Cd161+Ccr6+ T Cells Are Associated with Graft-Versus-Host Disease after Allogeneic Stem Cell Transplantation. *PLoS One* (2012) 7(12):e50896. Epub 2012/12/12. doi: 10.1371/journal.pone.0050896.

50. Khoder A, Sarvaria A, Alsuliman A, Chew C, Sekine T, Cooper N, et al. Regulatory B Cells Are Enriched within the Igm Memory and Transitional Subsets in Healthy Donors but Are Deficient in Chronic Gvhd. *Blood* (2014) 124(13):2034-45. Epub 2014/07/24. doi: 10.1182/blood-2014-04-571125.

51. Akı Ş Z, Suyanı E, Cengiz M, Özenirler S, Elbeğ Ş, Paşaoğlu H, et al. Association between Plasma Endothelin-1, Transforming Growth Factor-Β, Fibroblast Growth Factor, and Nitric Oxide Levels and Liver Injury in Hematopoietic Stem Cell Transplantation Recipients with Persistent Iron Overload after Transplantation. *Biology of blood and marrow transplantation : journal of the American Society for Blood and Marrow Transplantation* (2015) 21(5):948-53. Epub 2015/02/15. doi: 10.1016/j.bbmt.2015.02.002.

52. Allen JL, Fore MS, Wooten J, Roehrs PA, Bhuiya NS, Hoffert T, et al. B Cells from Patients with Chronic Gvhd Are Activated and Primed for Survival Via Baff-Mediated Pathways. *Blood* (2012) 120(12):2529-36. Epub 2012/08/17. doi: 10.1182/blood-2012-06-438911.

53. Brüggen MC, Petzelbauer P, Greinix H, Contassot E, Jankovic D, French L, et al. Epidermal Elafin Expression Is an Indicator of Poor Prognosis in Cutaneous Graft-Versus-Host Disease. *The Journal of investigative dermatology* (2015) 135(4):999-1006. Epub 2014/11/19. doi: 10.1038/jid.2014.489.

54. Cocho L, Fernández I, Calonge M, Martínez V, González-García MJ, Caballero D, et al. Biomarkers in Ocular Chronic Graft Versus Host Disease: Tear Cytokine- and Chemokine-Based Predictive Model. *Investigative ophthalmology & visual science* (2016) 57(2):746-58. Epub 2016/03/02. doi: 10.1167/iovs.15-18615.

55. Devic I, Shi M, Schubert MM, Lloid M, Izutsu KT, Pan C, et al. Proteomic Analysis of Saliva from Patients with Oral Chronic Graft-Versus-Host Disease. *Biology of blood and marrow transplantation : journal of the American Society for Blood and Marrow Transplantation* (2014) 20(7):1048-55. Epub 2014/04/08. doi: 10.1016/j.bbmt.2014.03.031.

56. Fedoriw Y, Samulski TD, Deal AM, Dunphy CH, Sharf A, Shea TC, et al. Bone Marrow B Cell Precursor Number after Allogeneic Stem Cell Transplantation and Gvhd Development. *Biology of blood and marrow transplantation : journal of the American Society for Blood and Marrow Transplantation* (2012) 18(6):968-73. Epub 2012/03/27. doi: 10.1016/j.bbmt.2012.03.005.

57. Gleimer M, Li Y, Braun TM, Chang L, Byersdorfer CA, Choi SW, et al. Baseline Body Mass Index among Children and Adults Undergoing Allogeneic Hematopoietic Cell Transplantation: Clinical Characteristics and Outcomes. *Bone Marrow Transplantation* (2015) 50(3):402-10. doi: <http://dx.doi.org/10.1038/bmt.2014.280>.

58. Knorr DA, Wang H, Aurora M, MacMillan ML, Holtan SG, Bergerson R, et al. Loss of T Follicular Helper Cells in the Peripheral Blood of Patients with Chronic Graft-Versus-Host Disease. *Biology of blood and marrow transplantation : journal of the American Society for Blood and Marrow Transplantation* (2016) 22(5):825-33. Epub 2016/01/26. doi: 10.1016/j.bbmt.2016.01.003.

59. Kuzmina Z, Greinix HT, Weigl R, Körmöczi U, Rottal A, Frantal S, et al. Significant Differences in B-Cell Subpopulations Characterize Patients with Chronic Graft-Versus-Host Disease-Associated Dysgammaglobulinemia. *Blood* (2011) 117(7):2265-74. Epub 2010/11/11. doi: 10.1182/blood-2010-07-295766.

60. Kuzmina Z, Gounden V, Curtis L, Avila D, Rnp TT, Baruffaldi J, et al. Clinical Significance of Autoantibodies in a Large Cohort of Patients with Chronic Graft-Versus-Host Disease Defined by Nih Criteria. *American journal of hematology* (2015) 90(2):114-9. Epub 2014/11/05. doi: 10.1002/ajh.23885.

61. Motta A, Zhan Q, Larson A, Lerman M, Woo SB, Soiffer RJ, et al. Immunohistopathological Characterization and the Impact of Topical Immunomodulatory Therapy in Oral Chronic Graft-Versus-Host Disease: A Pilot Study. *Oral diseases* (2018) 24(4):580-90. Epub 2017/12/03. doi: 10.1111/odi.12813.

62. Tumino M, Serafin V, Accordi B, Spadini S, Forest C, Cortese G, et al. Interleukin-22 in the Diagnosis of Active Chronic Graft-Versus-Host Disease in Paediatric Patients. *British journal of haematology* (2015) 168(1):142-5. Epub 2014/08/08. doi: 10.1111/bjh.13068.

63. Allen JL, Tata PV, Fore MS, Wooten J, Rudra S, Deal AM, et al. Increased Bcr Responsiveness in B Cells from Patients with Chronic Gvhd. *Blood* (2014) 123(13):2108-15. Epub 2014/02/18. doi: 10.1182/blood-2013-10-533562.

64. Budde H, Papert S, Maas JH, Reichardt HM, Wulf G, Hasenkamp J, et al. Prediction of Graft-Versus-Host Disease: A Biomarker Panel Based on Lymphocytes and Cytokines. *Annals of hematology* (2017) 96(7):1127-33. Epub 2017/04/28. doi: 10.1007/s00277-017-2999-5.

65. Babić A, Kurić L, Zelić Kerep A, Desnica L, Lelas A, Milošević M, et al. B Regulatory Cells and Monocyte Subpopulations in Patients with Chronic Graft-Vs-Host Disease. *Croatian medical journal* (2021) 62(2):154-64. Epub 2021/05/04. doi: 10.3325/cmj.2021.62.154.

66. Cocho L, Fernández I, Calonge M, Sainz de la Maza M, Rovira M, Stern ME, et al. Prehematopoietic Stem Cell Transplantation Tear Cytokines as Potential Susceptibility Biomarkers for Ocular Chronic Graft-Versus-Host Disease. *Investigative ophthalmology & visual science* (2017) 58(11):4836-46. Epub 2017/10/04. doi: 10.1167/iovs.17-21670.

67. Fingrut W, Law A, Lam W, Michelis FV, Viswabandya A, Lipton JH, et al. Post-Transplant Ferritin Level Predicts Outcomes after Allogeneic Hematopoietic Stem Cell Transplant, Independent from Pre-Transplant Ferritin Level. *Annals of Hematology* (2021) 100(3):789-98. doi: <http://dx.doi.org/10.1007/s00277-020-04363-1>.

68. Imanguli MM, Cowen EW, Rose J, Dhamala S, Swaim W, Lafond S, et al. Comparative Analysis of Foxp3(+) Regulatory T Cells in the Target Tissues and Blood in Chronic Graft Versus Host Disease. *Leukemia* (2014) 28(10):2016-27. Epub 2014/03/01. doi: 10.1038/leu.2014.92.

69. Whittle R, Taylor PC. Circulating B-Cell Activating Factor Level Predicts Clinical Response of Chronic Graft-Versus-Host Disease to Extracorporeal Photopheresis. *Blood* (2011) 118(24):6446-9. doi: <http://dx.doi.org/10.1182/blood-2011-05-354019>.

70. Mahadeo KM, Masinsin B, Kapoor N, Shah AJ, Abdel-Azim H, Parkman R. Immunologic Resolution of Human Chronic Graft-Versus-Host Disease. *Biology of blood and marrow transplantation : journal of the American Society for Blood and Marrow Transplantation* (2014) 20(10):1508-15. Epub 2014/07/01. doi: 10.1016/j.bbmt.2014.06.030.

71. Martires KJ, Baird K, Steinberg SM, Grkovic L, Joe GO, Williams KM, et al. Sclerotic-Type Chronic Gvhd of the Skin: Clinical Risk Factors, Laboratory Markers, and Burden of Disease. *Blood* (2011) 118(15):4250-7. Epub 2011/07/28. doi: 10.1182/blood-2011-04-350249.

72. Jung JW, Han SJ, Song MK, Kim TI, Kim EK, Min YH, et al. Tear Cytokines as Biomarkers for Chronic Graft-Versus-Host Disease. *Biology of blood and marrow transplantation : journal of the American Society for Blood and Marrow Transplantation* (2015) 21(12):2079-85. Epub 2015/08/26. doi: 10.1016/j.bbmt.2015.08.020.

73. Lynch Kelly D, Farhadfar N, Starkweather A, Garrett TJ, Yao Y, Wingard JR, et al. Global Metabolomics in Allogeneic Hematopoietic Cell Transplantation Recipients Discordant for Chronic Graft-Versus-Host Disease. *Biology of blood and marrow transplantation : journal of the American Society for Blood and Marrow Transplantation* (2020) 26(10):1803-10. Epub 2020/06/28. doi: 10.1016/j.bbmt.2020.06.014.

74. Grkovic L, Baird K, Steinberg SM, Williams KM, Pulanic D, Cowen EW, et al. Clinical Laboratory Markers of Inflammation as Determinants of Chronic Graft-Versus-Host Disease Activity and Nih Global Severity. *Leukemia* (2012) 26(4):633-43. Epub 2011/10/19. doi: 10.1038/leu.2011.254.

75. Kordelas L, Steckel NK, Horn PA, Beelen DW, Rebmann V. The Activating Nkg2c Receptor Is Significantly Reduced in Nk Cells after Allogeneic Stem Cell Transplantation in Patients with Severe Graft-Versus-Host Disease. *International journal of molecular sciences* (2016) 17(11). Epub 2016/11/02. doi: 10.3390/ijms17111797.

76. Marcondes AM, Li X, Tabellini L, Bartenstein M, Kabacka J, Sale GE, et al. Inhibition of Il-32 Activation by Α-1 Antitrypsin Suppresses Alloreactivity and Increases Survival in an Allogeneic Murine Marrow Transplantation Model. *Blood* (2011) 118(18):5031-9. Epub 2011/09/09. doi: 10.1182/blood-2011-07-365247.

77. Moins-Teisserenc H, Busson M, Herda A, Apete S, Peffault de Latour R, Robin M, et al. Cd19+Cd5+ B Cells and B1-Like Cells Following Allogeneic Hematopoietic Stem Cell Transplantation. *Biology of blood and marrow transplantation : journal of the American Society for Blood and Marrow Transplantation* (2013) 19(6):988-91. Epub 2013/03/20. doi: 10.1016/j.bbmt.2013.03.006.

78. de Masson A, Bouaziz JD, Le Buanec H, Robin M, O'Meara A, Parquet N, et al. Cd24(Hi)Cd27⁺ and Plasmablast-Like Regulatory B Cells in Human Chronic Graft-Versus-Host Disease. *Blood* (2015) 125(11):1830-9. Epub 2015/01/22. doi: 10.1182/blood-2014-09-599159.

79. Hirayama M, Azuma E, Nakagawa-Nakazawa A, Kumamoto T, Iwamoto S, Amano K, et al. Interleukin-10 Spot-Forming Cells as a Novel Biomarker of Chronic Graft-Versus-Host Disease. *Haematologica* (2013) 98(1):41-9. Epub 2012/06/27. doi: 10.3324/haematol.2012.069815.

80. Konuma T, Kohara C, Watanabe E, Takahashi S, Ozawa G, Suzuki K, et al. Reconstitution of Circulating Mucosal-Associated Invariant T Cells after Allogeneic Hematopoietic Cell Transplantation: Its Association with the Riboflavin Synthetic Pathway of Gut Microbiota in Cord Blood Transplant Recipients. *Journal of immunology (Baltimore, Md : 1950)* (2020) 204(6):1462-73. Epub 2020/02/12. doi: 10.4049/jimmunol.1900681.

81. Leonard JT, Newell LF, Meyers G, Hayes-Lattin B, Gajewski J, Heitner S, et al. Chronic Gvhd-Associated Serositis and Pericarditis. *Bone marrow transplantation* (2015) 50(8):1098-104. Epub 2015/05/12. doi: 10.1038/bmt.2015.105.

82. Sarvaria A, Basar R, Mehta RS, Shaim H, Muftuoglu M, Khoder A, et al. Il-10+ Regulatory B Cells Are Enriched in Cord Blood and May Protect against Cgvhd after Cord Blood Transplantation. *Blood* (2016) 128(10):1346-61. Epub 2016/07/22. doi: 10.1182/blood-2016-01-695122.

83. Botari CM, Nunes AJ, Souza MP, Orti-Raduan ES, Salvio AG. Oral Chronic Graft-Versus-Host Disease: Analysis of Dendritic Cells Subpopulations. *Anais brasileiros de dermatologia* (2014) 89(4):632-7. Epub 2014/07/24. doi: 10.1590/abd1806-4841.20142464.

84. Duraković N, Krečak I, Perić Z, Milošević M, Desnica L, Pulanić D, et al. Glycoprotein Ykl-40: A Novel Biomarker of Chronic Graft-Vs-Host Disease Activity and Severity? *Croatian medical journal* (2016) 57(3):239-46. Epub 2016/07/05. doi: 10.3325/cmj.2016.57.239.

85. Eberwein P, Issleib S, Böhringer D, Mittelviefhaus H, Schwartzkopff J, Finke J, et al. Conjunctival Hla-Dr and Cd8 Expression Detected by Impression Cytology in Ocular Graft Versus Host Disease. *Molecular vision* (2013) 19:1492-501. Epub 2013/07/24.

86. Konuma T, Kohara C, Watanabe E, Mizukami M, Nagai E, Oiwa-Monna M, et al. Circulating Monocyte Subsets in Human Chronic Graft-Versus-Host Disease. *Bone marrow transplantation* (2018) 53(12):1532-40. Epub 2018/05/08. doi: 10.1038/s41409-018-0187-4.

87. Chasset F, de Masson A, Le Buanec H, Xhaard A, de Fontbrune FS, Robin M, et al. April Levels Are Associated with Disease Activity in Human Chronic Graft-Versus-Host Disease. *Haematologica* (2016) 101(7):e312-5. Epub 2016/03/20. doi: 10.3324/haematol.2016.145409.

88. Lai P, Weng J, Lu Z, Guo R, Luo C, Wu S, et al. Gene Expression Profiling-Based Identification of Cd28 and Pi3k as New Biomarkers for Chronic Graft-Versus-Host Disease. *DNA and cell biology* (2011) 30(12):1019-25. Epub 2011/06/21. doi: 10.1089/dna.2011.1284.

89. Hu Y, Cui Q, Ye Y, Luo Y, Tan Y, Shi J, et al. Reduction of Foxp3+ T Cell Subsets Involved in Incidence of Chronic Graft-Versus-Host Disease after Allogeneic Hematopoietic Stem Cell Transplantation. *Hematological oncology* (2017) 35(1):118-24. Epub 2015/10/07. doi: 10.1002/hon.2255.

90. Hirayama M, Azuma E, Iwamoto S, Amano K, Nakazawa A, Tamaki S, et al. High Frequency of Cd29high Intermediate Monocytes Correlates with the Activity of Chronic Graft-Versus-Host Disease. *European journal of haematology* (2013) 91(3):280-2. Epub 2013/06/26. doi: 10.1111/ejh.12160.

91. St John L, Gordon SM, Childs R, Marquesen M, Pavletic SZ, Wu TX, et al. Topical Thalidomide Gel in Oral Chronic Gvhd and Role of in Situ Cytokine Expression in Monitoring Biological Activity. *Bone marrow transplantation* (2013) 48(4):610-1. Epub 2012/09/25. doi: 10.1038/bmt.2012.178.
